# Identification of a molecular resistor that controls UCP1-independent Ca^2+^ cycling thermogenesis in adipose tissue

**DOI:** 10.1016/j.cmet.2025.03.009

**Published:** 2025-06-03

**Authors:** Christopher Auger, Mark Li, Masanori Fujimoto, Kenji Ikeda, Jin-Seon Yook, Timothy R. O’Leary, María Paula Huertas Caycedo, Cai Xiaohan, Satoshi Oikawa, Anthony R.P. Verkerke, Kosaku Shinoda, Patrick R. Griffin, Kenji Inaba, Roland H. Stimson, Shingo Kajimura

**Affiliations:** 1 Division of Endocrinology, Diabetes and Metabolism, Beth Israel Deaconess Medical Center and Harvard Medical School, and Howard Hughes Medical Institute, Boston, MA, USA; 2 Department of Molecular Endocrinology and Metabolism, Tokyo Medical and Dental University, Tokyo, Japan; 3 Department of Molecular Medicine, The Herbert Wertheim UF Scripps Institute for Biomedical Innovation & Technology, Jupiter, FL, USA; 4 University/BHF Centre for Cardiovascular Science, University of Edinburgh, Queen’s Medical Research Institute, Edinburgh, UK; 5 Medical Institute of Bioregulation, Kyushu University, Fukuoka 812-8582, Japan; 6 Core Research for Evolutional Science and Technology (CREST), Japan Agency for Medical Research and Development (AMED), Tokyo, Japan; 7 Departments of Medicine and Molecular Pharmacology, Albert Einstein College of Medicine, Bronx, New York, NY, USA

**Keywords:** thermogenesis, UCP1-independent, Ca^2+^ cycling, energy balance, obesity

## Abstract

Adipose tissue thermogenesis contributes to energy balance via mitochondrial uncoupling protein 1 (UCP1) and UCP1-independent pathways. Among UCP1-independent thermogenic mechanisms, one involves Ca^2+^ cycling via SERCA2b in adipose tissue; however, the underlying molecular basis remains elusive. Here, we report that an endoplasmic reticulum (ER) membrane-anchored peptide, C4orf3 (also known as another regulin [ALN]), uncouples SERCA2b Ca^2+^ transport from its ATP hydrolysis, rendering the SERCA2b-C4orf3 complex exothermic. Loss of C4orf3/ALN improved the energetic efficiency of SERCA2b-dependent Ca^2+^ transport without affecting SERCA2 expression, thereby reducing adipose tissue thermogenesis and increasing the adiposity of mice. Notably, genetic depletion of *C4orf3* resulted in compensatory activation of UCP1-dependent thermogenesis following cold challenge. We demonstrated that genetic loss of both *C4orf3* and *Ucp1* additively impaired cold tolerance *in vivo*. Together, this study identifies C4orf3 as the molecular resistor to SERCA2b-mediated Ca^2+^ import that plays a key role in UCP1-independent thermogenesis and energy balance.

## Highlights

• Development of a platform that directly records microsomal thermogenesis
• C4orf3 promotes Ca^2+^ cycling thermogenesis by reducing SERCA2 energetic efficiency
• C4orf3 is required for UCP1-independent thermogenesis *in vivo*
• Loss of C4orf3 resulted in increased adiposity and systemic insulin resistance

## Graphical abstract

**Figure:**
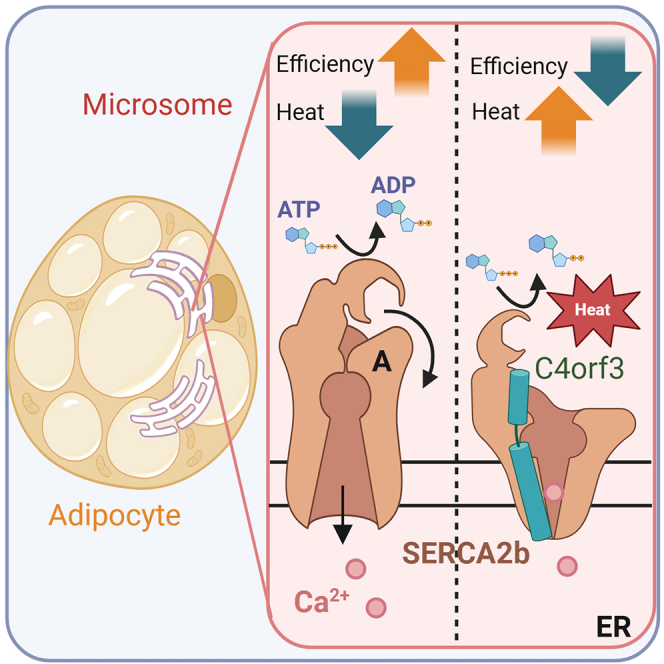

## Teaser

Adipose tissue contributes to energy balance via uncoupling protein 1 (UCP1) and UCP1-independent thermogenic pathways. One of the UCP1-independent mechanisms involves Ca^2+^ futile cycling, but the underlying mechanism remains elusive. Auger et al. identify C4orf3/ALN as a “molecular resistor” to SERCA2b-mediated Ca^2+^ import, thereby controlling UCP1-independent thermogenesis and energy homeostasis.

## Introduction

The principle of thermogenesis follows Joule’s first law: Q (J) = *I*^2^ × R × *T*, where *I* is current, *R* is resistance, and *T* is time.^1^ This formula explains how proton uncoupling of the mitochondrial electron transport chain (ETC) by uncoupling protein 1 (UCP1) leads to thermogenesis in brown and beige adipocytes. For over three decades, UCP1 was thought to be the only protein responsible for non-shivering thermogenesis in mammals.^2^^,^^3^ Accordingly, the prevailing theory for the metabolic benefits associated with active brown/beige fat was primarily through UCP1-dependent thermogenesis.^4^ However, many mammalian species, including pigs, lack the functional *UCP1* gene,^5^^,^^6^ yet pig adipocytes retain thermogenic capacity.^7^ Furthermore, marsupials possess beige-like adipose tissue and maintain body temperature, even though their UCP1 is non-thermogenic.^8^ Importantly, emerging evidence shows that adipose tissue generates heat through UCP1-independent mechanisms, a.k.a. futile cycling thermogenesis, which contribute to whole-body energy balance.^7^^,^^9^^,^^10^^,^^11^

Recent studies provided mechanistic insights into UCP1-independent thermogenesis in adipose tissue.^12^^,^^13^^,^^14^ For example, futile creatine cycling involves ATP-dependent creatine phosphorylation by creatine kinase B (CKB) and its dephosphorylation by the mitochondria-localized TNAP.^11^^,^^15^^,^^16^ Lipid futile cycling leads to thermogenesis when adipocyte lipolysis and fatty acid re-esterification are simultaneously activated.^17^^,^^18^ Ca^2+^ cycling generates heat when active Ca^2+^ uptake into the endoplasmic/sarcoplasmic reticulum (ER)/SR lumen occurs through the action of the ATP-dependent Ca^2+^ pump SERCA1 in the skeletal muscle^19^ or SERCA2b in adipocytes,^7^^,^^20^ while Ca^2+^ is simultaneously released into the cytosolic compartment through inositol trisphosphate receptors (IP3Rs) and ryanodine receptors (RYRs). The common feature of these UCP1-independent pathways is ATP-linked substrate futile cycling, in which two opposing metabolic pathways run simultaneously without any overall work effect.^12^^,^^13^ Futile cycling pathways are tightly regulated by physiological stimuli, such as norepinephrine (NE), whereas dysregulation can lead to pathological hyperthermia. For instance, genetic mutations in the *RYR1* gene (the skeletal muscle form) cause Ca^2+^ leak from the SR compartment, resulting in malignant hyperthermia in humans and also in pigs.^21^^,^^22^

Exactly how ATP-linked futile cycling generates heat remains insufficiently understood. Following Joule’s first law, the crux of understanding UCP1-independent futile cycling thermogenesis is to identify the molecular “resistor” (the factor *R*) in an ATP-linked reaction. In this context, sarcolipin (SLN) provides resistance to Ca^2+^ cycling in skeletal muscle by binding to SERCA1 and reducing its Ca^2+^ transport activity, facilitating muscle thermogenesis.^23^^,^^24^ However, mouse subcutaneous adipose tissue does not express SLN or other known SERCA1-binding peptides, including phospholamban (PLN), myoregulin (MLN), and DWORF^25^^,^^26^^,^^27^ (Figure S1A). Accordingly, we set forth to identify the molecular resistance to SERCA2b-dependent Ca^2+^ cycling in adipose tissue.

A technical hurdle in addressing this question is that measuring oxygen consumption rate (OCR) does not always reflect the total amount of heat produced in a reaction when *R* (resistance) is minimal: e.g., cardiomyocytes have high OCR but low thermogenic capacity due to low *R*. To overcome this hurdle, we have developed a platform to quantify heat production at organelle resolution using isothermal titration calorimetry (ITC). This approach allows us to directly record thermogenesis in isolated microsomes from tissues and cultured cells.

## Results

### Direct recording of microsomal thermogenesis in adipose tissue

**Figure 1:**
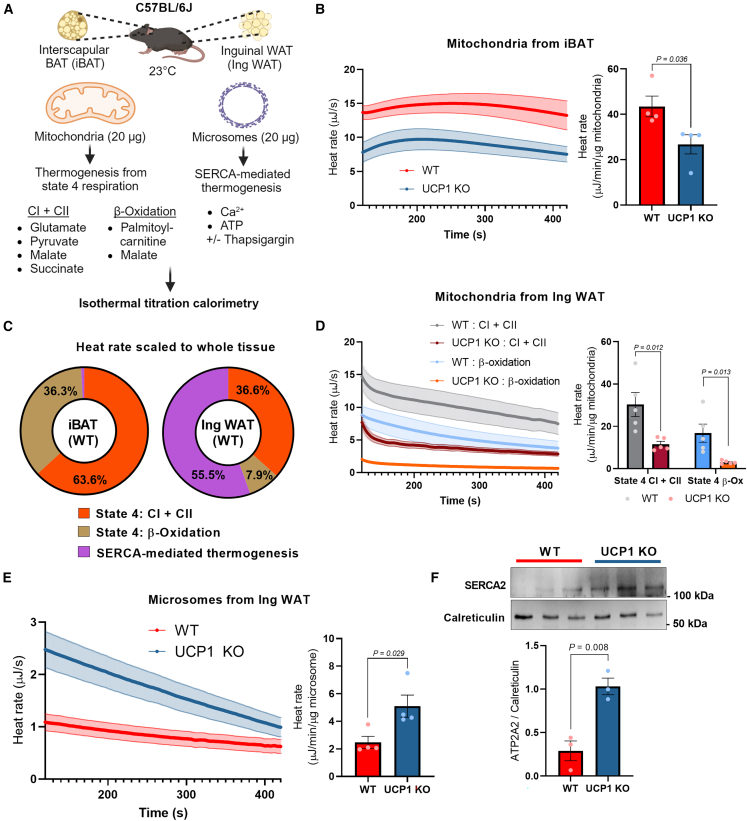
Direct recording of microsomal thermogenesis in adipose tissue (A) Schematic of organelle thermogenesis assays using ITC. Isolated mitochondria (20 μg) were subjected to ITC in the presence of glutamate, pyruvate, malate, and succinate (complexes I and II) or palmitoyl-carnitine and malate (β-oxidation). To detect microsomal thermogenesis, isolated microsomes (20 μg) were subjected to ITC in the presence of ATP at 1 mM and free Ca^2+^ at 10 μM. Thapsigargin was added to determine SERCA-dependent signals. (B) Heat rate (μJ s^−1^) in isolated mitochondria from the iBAT of wild-type (WT) and UCP1 KO male mice following the addition of palmitoyl-carnitine and malate. Heat rate was normalized by mitochondrial protein contents (μJ min^−1^ μg^−1^). *n* = 4. (C) Contributions of mitochondrial vs. microsomal thermogenesis in the iBAT and inguinal WAT. Mitochondrial thermogenesis was measured in the presence of glutamate, pyruvate, malate, and succinate (complexes I + II) or in the presence of palmitoyl-carnitine and malate (β-oxidation). Thapsigargin-independent heat rate in microsomes was excluded as background. *n* = 4. (D) Heat rate in isolated mitochondria from IngWAT of WT and UCP1 KO male mice. Mitochondrial thermogenesis (complexes I + II or β-oxidation) was measured and normalized by mitochondrial protein contents (μJ min^−1^ μg^−1^). *n* = 5. (E) Heat rate in isolated microsomes from the inguinal WAT of WT and UCP1 KO male mice in the presence of ATP and free Ca^2+^ (pCa 6.0). The heat rate was normalized by microsome protein contents (μJ min^−1^ μg^−1^). *n* = 4. (F) Immunoblotting of SERCA2 and ER-localized protein calreticulin as a loading control in the isolated microsomes from WT and UCP1 KO male mice. *n* = 3. Statistic (B and D–F): unpaired t test. Bars represent the mean and error shown as SEM.

We employed a high-resolution ITC instrument with a minimum heat detection of 0.04 μJ to detect heat production in the isolated mitochondrial and microsomal compartments from the interscapular brown adipose tissue (iBAT) and the inguinal white adipose tissue (IngWAT) of mice (Figure 1A). In the isolated mitochondria, we measured heat production following the addition of malate, pyruvate, glutamate, and succinate to activate complex I + II, or the addition of palmitoyl-carnitine and malate to activate mitochondrial β-oxidation. In the isolated microsomes, we recorded heat production in the microsomal fraction in response to ATP (1 mM) and free Ca^2+^ in the presence or absence of thapsigargin, a non-competitive pan SERCA inhibitor. To determine SERCA-dependent thermogenesis, microsomal heat rates in the presence of thapsigargin were subtracted as background from those measured without thapsigargin in each sample. To exclude heat production arising from non-specific ion bindings, we recorded heat production in the microsomes starting 2 min after the reaction and continuing for 5 min following the previous protocol^28^ (Figure S1B). Note that no heat production was detected in samples without Ca^2+^ or in the presence of the non-hydrolyzable ATP analog AMP-PNP and Ca^2+^ (Figure S1C). These data validated the specificity of the ITC-based thermogenesis assay in isolated organelles.

To validate the assay, we next examined mitochondrial thermogenesis in the iBAT of wild-type control mice and UCP1 knockout (KO) mice. The ITC-based measurement detected significant amounts of heat that were dependent on UCP1, as 38.5% of heat was lost in the iBAT mitochondria of UCP1 KO mice (Figure 1B). Heat production in UCP1 KO mitochondria would presumably occur through UCP1-independent thermogenesis and substrate reactions in the matrix. Note that the assays were performed in isolated mitochondria with identical amounts of mitochondrial protein. On the other hand, we found no detectable SERCA-dependent heat production (i.e., thapsigargin-sensitive exothermic reaction) in the microsomal fraction from the iBAT (Figure S1D). This result was consistent with our previous work showing that Ca^2+^ cycling thermogenesis was selective for the inguinal WAT but negligible in the iBAT.^7^ To that effect, mitochondrial thermogenesis predominantly drives the overall heat production in iBAT (Figure 1C). This might be due to the fact that brown adipocytes in the iBAT contain low amounts of ATP synthase and have a limited capacity to generate ATP^29^; hence, the contribution of ATP-dependent thermogenesis in the microsomes would be less in the iBAT. In stark contrast to iBAT, we detected significant amounts of SERCA-dependent heat in isolated microsome fractions from the inguinal WAT of wild-type mice. The microsomal heat production was equivalent to or even higher than the mitochondria-derived heat production in the inguinal WAT (55.5% of the total heat measured), even though the total amount of heat generated in inguinal WAT was substantially lower than that in iBAT. It is important to note that microsomal thermogenesis in inguinal WAT was highly SERCA2-dependent because the heat production was largely diminished in the presence of thapsigargin (Figure S1E). Similarly, microsomal thermogenesis was lost in the inguinal WAT of adipocyte-specific SERCA2 KO mice that we developed previously^7^ (Figure S1F).

With this platform in hand, we asked whether loss of UCP1 triggers a compensatory activation of Ca^2+^ cycling thermogenesis. We isolated the microsomal and mitochondrial fractions from the inguinal WAT of wild-type mice and UCP1 KO mice that were chronically treated with the β3-adrenergic receptor agonist CL316,243 for 5 days. In the mitochondrial fraction, ITC-based heat measurement showed a decrease in UCP1-deficient mitochondria, suggesting that UCP1-dependent thermogenesis mediates a large part of the mitochondrial thermogenesis in the inguinal WAT (Figure 1D). On the other hand, ITC-based thermogenesis assays at pCa 6.0 found a significant increase in microsomal thermogenesis in the inguinal WAT of UCP1 KO mice (Figure 1E). This increase in microsomal thermogenesis was accompanied by an upregulation of SERCA2 protein expression in the inguinal WAT (Figure 1F). Together, these data suggest that UCP1-independent Ca^2+^ cycling thermogenesis contributes to the overall heat production in the inguinal WAT, particularly in the absence of UCP1.

### C4orf3 promotes Ca^2+^ cycling thermogenesis by reducing the energetic efficiency of SERCA2

**Figure 2:**
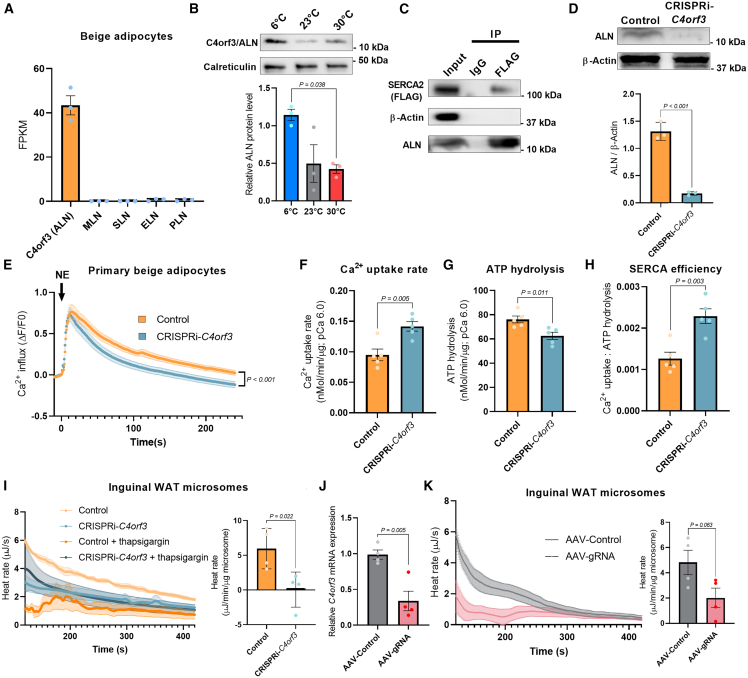
C4orf3 promotes Ca^2+^ cycling thermogenesis by reducing the energetic efficiency of SERCA2-dependent Ca^2+^ transport (A) FPKM of indicated SERCA-binding peptides in isolated beige adipocytes of mice. *n* = 3. (B) Relative C4orf3/ALN protein expression levels in microsomes from IngWAT of UCP1 KO mice at the corresponding temperature. Calreticulin was used as a loading control. *n* = 3. Statistic: one-way ANOVA with Tukey’s post hoc HSD test. (C) Protein interaction between SERCA2b and endogenous C4orf3/ALN protein in beige adipocytes. Immunoprecipitants of a SERCA2 complex (FLAG-tagged) were immunoblotted using a polyclonal antibody for C4orf3. Inputs were included in the immunoblotting. (D) Endogenous C4orf3 protein expression in IngWAT of *C4orf3*^CRISPRi^ mice and control male mice. β-actin was used as a loading control. *n* = 3. (E) Intracellular Ca^2+^ flux assay in IngWAT-derived adipocytes from control and *C4orf3*^CRISPRi^ mice. Primary adipocytes were differentiated on collagen-coated glass-bottom dishes in which intercellular Ca^2+^ levels were determined by using the Fluo-8 dye. Control, *n* = 50; *C4orf3*^CRISPRi^, *n* = 50. Statistic: two-way ANOVA with Šídák’s multiple comparisons test. (F) Ca^2+^ uptake in isolated microsomes from the inguinal WAT of male *C4orf3*^CRISPRi^ mice and control mice at pCa^2+^ 6.0. *n* = 5. (G) SERCA ATP hydrolysis assay in isolated microsomes from (F). (H) The bioenergetic efficiency of SERCA2 was calculated by Ca^2+^ uptake per ATP hydrolysis from (E) and (F). (I) Heat rate in isolated microsomes from IngWAT of control and *C4orf3*^CRISPRi^ male mice in the presence of ATP and Ca^2+^ (pCa 6.0). Thapsigargin was added to calculate SERCA-dependent thermogenesis. *n* = 5. (J) Relative mRNA levels of *C4orf3* in adult dCas9-KRAB male mice 2 weeks after direct injection into IngWAT of AAV expressing scrambled control or gRNA targeting *C4orf3*. *n* = 4. (K) Heat rate in isolated microsomes from the inguinal WAT of dCas9-KRAB male mice 2 weeks after AAV injection into the inguinal WAT. *n* = 4. Statistic (D and F–K): unpaired t test. Bars represent the mean and error shown as SEM.

Our transcriptomics data^7^ found undetectable levels of known SERCA1-binding peptides, including SLN in mouse adipose tissue (Figure S1A). Thus, we searched for adipocyte-enriched factors that control Ca^2+^ cycling thermogenesis by binding to the SERCA2b protein. To this end, we performed the following bioinformatic analyses: first, we mapped long non-coding RNAs (lncRNAs) that were significantly expressed (TPM > 10) in the inguinal adipose tissue of fat-selective PRDM16 transgenic × *Ucp1* KO mice in which we previously found enhanced Ca^2+^ cycling thermogenesis.^7^ Second, we examined the homology between the predicted amino acid sequences of the above-identified lncRNAs and SERCA1-binding peptides, including PLN and SLN. We then generated 3D structural models and predicted the interaction between these candidates and SERCA proteins by using the HDOCK server.^30^ These analyses identified 5 candidates (Figure S2A). Based on the structural homology to known peptides and the binding prediction, the top candidate was C4orf3, which encodes a 65-amino-acid peptide. C4orf3 was previously reported by the Olson lab and termed another regulin (ALN)^27^ because of its similar inhibitory effect on SERCA’s Ca^2+^ transport to PLN. We confirmed in independent samples that C4orf3/ALN mRNA was highly expressed in beige adipocytes, whereas other SERCA-binding peptides (MLN, SLN, PLN, and DWORF) were not detected (Figure 2A). Single-cell RNA sequencing (RNA-seq) data analyses of mouse inguinal WAT from a recent study^31^ showed that genes involved in Ca^2+^ cycling, including *Atp2a2* (SERCA2), *Itpr1*, and *Itpr2*, were enriched in P2 and P5 beige adipocyte populations, although other populations also express these genes at lower levels. *C4orf3* was expressed in P2, P5, P7, and other populations except P6 and P9, albeit at low levels (Figure S2B). It is notable as the P2 beige adipocyte population is shown to express *Atp5k* with high ATP synthesis capacity and utilize ATP-linked futile cycle thermogenesis.^31^ We then developed a polyclonal antibody against C4orf3/ALN and found that the endogenous protein was highly expressed in the inguinal WAT and several other tissues, including the heart, kidney, and brain, but nearly undetectable in skeletal muscle (Figure S2C). C4orf3/ALN protein expression in the inguinal WAT of UCP1 KO cold-acclimated mice was higher than that of warm-acclimated mice at 30°C (Figure 2B). Furthermore, we validated the protein interaction between SERCA2b and the endogenous C4orf3/ALN protein in inguinal WAT-derived differentiated adipocytes (Figure 2C).

Previous studies showed that overexpression of C4orf3/ALN inhibits SERCA2-mediated Ca^2+^ transport in cultured cells, similar to other known SERCA-binding peptides.^27^^,^^32^ However, the biological role of C4orf3/ALN *in vivo* remains unknown. Thus, we generated mice lacking *C4orf3* by employing the CRISPRi system in which the catalytically inactive Cas9 protein (dCas9) was fused to Krüppel-associated box (KRAB) domain.^33^ Transgenic mice expressing an effective gRNA targeting *C4orf3* were crossed with dCas9-KRAB mice to generate C4orf3/ALN*-*deficient mice (CRISPRi-*C4orf3*) (Figures 2D and S2D). Subsequently, we harvested the stromal vascular fraction (SVF) from the inguinal WAT of CRISPRi-*C4orf3* mice and control mice. In differentiated adipocytes from the primary SVF, we measured intracellular Ca^2+^ flux in response to NE, a potent thermogenic stimulus. We found that NE rapidly elevated intracellular Ca^2+^ levels both in control and C4orf3/ALN-depleted adipocytes. However, C4orf3/ALN-depleted adipocytes exhibited faster sequestration of intracellular Ca^2+^ than control adipocytes (Figure 2E). This observation was further validated *in vivo*: ^45^Ca uptake in the microsomes isolated from inguinal WAT of CRISPRi-*C4orf3* mice was significantly higher than that in littermate control mice at pCa 6.0 (Figure 2F). These results suggest that C4orf3/ALN-depleted adipocytes uptake Ca^2+^ into the ER compartment more rapidly than control adipocytes. This is in agreement with the previous gain-of-function studies in cultured cells that C4orf3/ALN is “another” inhibitory regulator of SERCA like other SERCA1-binding peptides.^27^^,^^32^

However, the following data suggest that C4orf3/ALN has a distinct functional property from other inhibitory peptides. We found that C4orf3 depletion modestly but significantly reduced the ATP hydrolysis activity of SERCA2 in the adipose tissue by 17.8% (Figures 2G and S2E). This is in contrast to other inhibitory peptides that reduce both SERCA ATP hydrolysis and Ca^2+^ transport activities (note: if C4orf3/ALN were solely an inhibitory peptide, SERCA2’s ATP hydrolysis would be increased in *C4orf3* KO mice). When we calculated the efficiency of Ca^2+^ import *per* ATP hydrolysis, we found that C4orf3/ALN-depleted adipose tissues exhibited substantially higher Ca^2+^ transport efficiency than control adipose tissues by 90.6% (Figure 2H). It is worth noting that there was no difference in SERCA2 mRNA and protein expression levels in the inguinal WAT between CRISPRi-*C4orf3* mice and control mice (Figures S2F and S2G). These results suggest that C4orf3/ALN binding to SERCA2 alters the stoichiometry of ATP hydrolysis to Ca^2+^ transport (i.e., coupling efficiency) rather than purely being an inhibitory peptide or altering SERCA2 expression. The change in Ca^2+^ transport efficiency reflects the *R* factor (resistance) in Joule’s law.

To test if the C4orf3/ALN-SERCA2 complex alters Ca^2+^ cycling thermogenesis, we employed ITC-based thermogenic assays to measure the amount of heat production in the microsomal fractions from the inguinal WAT of CRISPRi-*C4orf3* mice and littermate controls at pCa 6.0. We found that the microsomes from CRISPRi-*C4orf3* mice produced substantially less heat than those of control mice (Figure 2I). The difference between the two groups was SERCA2-dependent thermogenesis, as ascertained by measurements taken in the presence of the SERCA inhibitor thapsigargin. This result is consistent with the data that C4orf3 changes Ca^2+^ transport efficiency (i.e., low *R*), and thus, depletion of C4orf3 resulted in reduced SERCA2-dependent Ca^2+^ cycling thermogenesis. On the other hand, there was no statistical difference in the microsomal thermogenesis in the iBAT, liver, and kidney between the two groups, although there was a modest trend in the kidney (Figure S2H).

To determine the tissue-selective role of ALN on microsomal thermogenesis further, we next injected AAV-expressing gRNA targeting *C4orf3* or control AAV in the inguinal WAT of dCas9-KRAB mice. AAV-gRNA administration successfully reduced *C4orf3* mRNA expression by 66.6% relative to control AAV (Figure 2J). Consistent with the observation made in whole-body CRISPRi-*C4orf3* mice, we found that microsomal thermogenesis in the inguinal WAT of AAV-gRNA-treated mice was reduced relative to control mice (Figure 2K). Taken together, these results indicate the unique role of C4orf3/ALN on Ca^2+^ cycling thermogenesis in the inguinal WAT.

### A structural mechanism by which C4orf3 alters the Ca^2+^ transport efficiency of SERCA2

**Figure 3:**
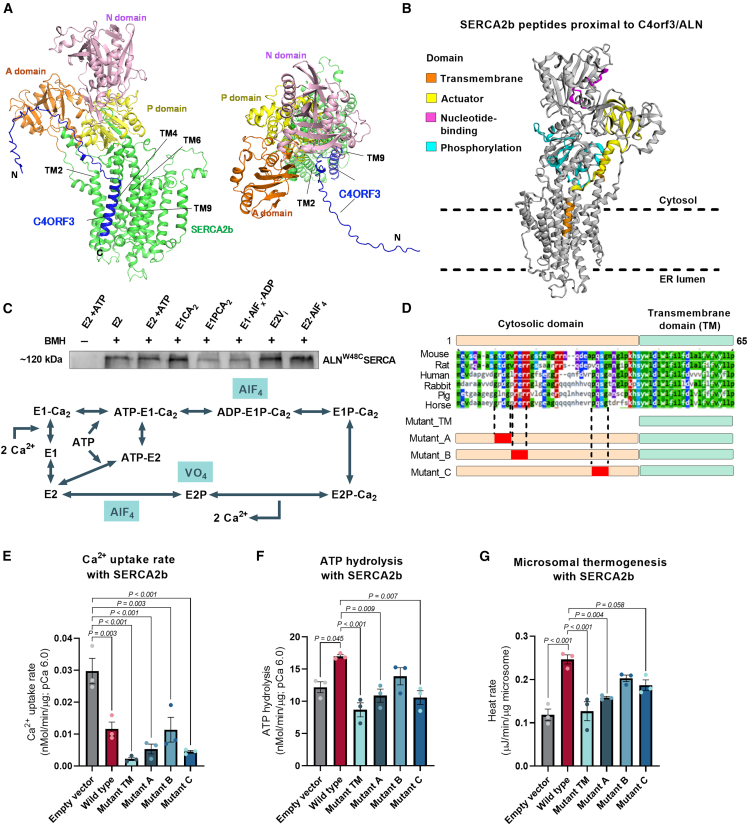
A structural mechanism by which C4ORF3 alters the Ca^2+^ transport efficiency of SERCA2 (A) Side (left) and top (right) views of the SERCA2b-C4ORF3 complex structure predicted by AlphaFold3. The A-, N-, P-, and TM domains of Ca^2+^-unbound SERCA2b are shown in orange, pink, yellow, and green, respectively. C4ORF3/ALN is shown in blue. (B) Protein interaction interface of SERCA2b to C4orf3 as determined by the Turbo-ID proximity-labeling proteomics. Turbo-tag was fused to the N terminus (the cytoplasmic side) of C4orf3. Orange, TM domain; yellow, actuator domain; pink, nucleotide-binding domain; cyan, P-domain. (C) Cross-linking assays of SERCA2b with C4orf3/ALN^W48C^ in isolated microsomes at indicated SERCA2 catalytic states. The cross-linker BMH was added to induce the SERCA2-C4ORF3 complex formation. The assays were performed in three independent biological samples. Bottom: catalytic cycle of SERCA to transport Ca^2+^ into the ER lumen. Indicated compounds were used to induce the specific catalytic states of SERCA. (D) The amino acid sequence alignment of C4ORF3. The lower panel shows the schematics of a C4ORF3 mutant containing only the transmembrane (TM) domain and three mutants (mutants A, B, and C) lacking the evolutionarily conserved domains (shown in red boxes). (E) Ca^2+^ uptake in isolated microsomes from cells co-expressing SERCA2b together with empty vector control and indicated C4ORF3 constructs at pCa^2+^ 6.0. *n* = 3. (F) SERCA ATP hydrolysis assay in microsomes from (E). (G) ITC-based thermogenesis assays from microsomes in (E). Statistic (E–G): one-way ANOVA with Tukey’s post hoc HSD test. Bars represent the mean and error shown as SEM.

The SERCA protein undergoes dynamic structural changes to transport cytosolic Ca^2+^ into the ER/SR lumen side during the catalytic cycle.^34^^,^^35^^,^^36^ During the transition from the E1⋅ 2Ca^2+^ state to the E2 state, the actuator domain (A-domain) of SERCA2 rotates by nearly 100°, while the transmembrane (TM) helix cluster TM1–4 of SERCA2 opens to release Ca^2+^ into the luminal side.^37^^,^^38^ Nevertheless, it remains unclear how C4orf3/ALN controls the Ca^2+^ transport efficiency of SERCA2b. In contrast to SLN, which binds to SERCA1 exclusively at the intramembrane domain,^39^ C4orf3/ALN has an unusually long extramembrane domain (Figure S3A). AlphaFold3-based prediction suggests that the TM domain of C4orf3/ALN binds to the TM groove formed by TM2, TM4, TM6, and TM9 of SERCA2b as similar to SLN in the complex with SERCA1.^39^^,^^40^^,^^41^ On the other hand, the cytoplasmic domain of C4orf3/ALN is in close proximity to the phosphorylation domain (P-domain) of SERCA2b (Figure 3A).

To validate this prediction experimentally, we next mapped the binding interface of C4orf3/ALN and SERCA2b by employing Turbo-ID proximity labeling proteomics.^42^ We expressed C4orf3/ALN that was fused to a Turbo-ID proximity-labeling tag at the N terminus (the cytoplasmic side of C4orf3/ALN) in beige adipocytes. C4orf3/ALN-associated proteins were detected by biotinylated peptides using Turbo-ID, followed by tandem mass tag (TMT)-based quantitative proteomics. This experiment identified four peptides from the P-domain and one peptide from TM2 of SERCA2b, which are in agreement with the AlphaFold3-predicted structure (Figure 3B). We also identified peptides from the A- and N-domains, suggesting the high flexibility of the C4orf3/ALN cytosolic domain in the complex with SERCA2b. Intriguingly, we detected identical SERCA2b peptides when the Turbo-ID proximity labeling was performed in beige adipocytes treated with NE (Figure S3B). The data suggest that C4orf3/ALN constitutively interacts with SERCA2b in the basal and NE-stimulated states. In line with this result, cross-linking experiments using isolated microsomes showed that C4orf3/ALN interacts with SERCA2b throughout the catalytic cycle of SERCA2b, although C4orf3/ALN tends to bind SERCA2b in the E2 state more stably than in the E1 state (Figures 3C and S3C). A similar state-dependent binding between SERCA1 and SLN was previously observed in microsomes in which SLN decreased SERCA1-dependent Ca^2+^ uptake without affecting its ATP hydrolysis.^23^

Given the long cytoplasmic domain that is unique to C4orf3/ALN, we next asked whether this domain is functionally required for SERCA2-dependent Ca^2+^ cycling thermogenesis. To this end, we generated a truncated mutant lacking the cytoplasmic domain (mutant TM). In addition, we generated three C4orf3 mutants (mutants A, B, and C) with amino acid substitutions in three evolutionarily conserved segments of the cytoplasmic region (Figures 3D and S3D). Full-length (wild-type) C4orf3 and these mutants were then ectopically expressed together with SERCA2b in HEK293 cells (Figure S3E). We found that all the constructs significantly decreased the Ca^2+^ transport rate of SERCA2 (Figure 3E). The mutant lacking the cytoplasmic domain (mutant TM) potently reduced the SERCA2-dependent Ca^2+^ transport rate, even though its protein expression level was lower than that of the wild type. These results suggest that the inhibitory effect of C4orf3/ALN on SERCA2 Ca^2+^ transport is mediated by the SERCA2-C4orf3/ALN interaction at the TM domain. By contrast, wild-type C4orf3/ALN significantly activated SERCA2 ATP hydrolysis, and this action required its cytoplasmic domain. Mutants A and C did not alter the ATPase activity of SERCA2 (Figure 3F), suggesting that segments A and C in the cytoplasmic region of C4orf3/ALN are necessary for the action of C4orf3/ALN on activating SERCA2 ATP hydrolysis. Importantly, ITC-based thermogenesis assays in purified microsomes from these cells found that full-length C4orf3/ALN potently stimulated microsomal thermogenesis, whereas C4orf3 mutants lacking the cytoplasmic domain (mutant TM) or the domain A/C (mutants A and C) failed to stimulate microsomal thermogenesis (Figures 3G and S3F). The effect of C4orf3/ALN on microsomal thermogenesis was dependent on SERCA2 because ectopic expression of ALN and mutants did not affect microsomal thermogenesis in cells lacking SERCA2 (Figure S3G). We also note that overall heat production from the microsomes of cultured HEK293 cells was markedly lower than that of inguinal WAT, possibly owing to changes in ER membrane composition or other modulators of Ca^2+^ cycling. These results support the AlphaFold3-predicted structure of the SERCA2b-C4orf3/ALN complex in which the cytoplasmic region of C4orf3/ALN partly interacts with the SERCA2b P-domain that is in charge of ATP hydrolysis (Figure 3A). Together, C4orf3/ALN facilitates microsomal Ca^2+^ cycling thermogenesis by decreasing the Ca^2+^ transport efficiency of SERCA2 via the interaction at the TM domain of C4orf3/ALN, while increasing its ATPase activity through the cytoplasmic region.

### C4orf3/ALN is required for UCP1-independent thermogenesis *in vivo*

**Figure 4:**
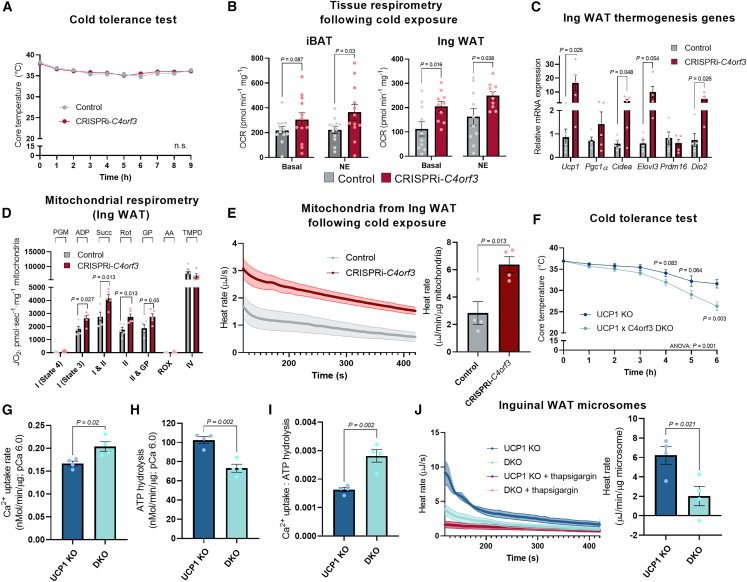
C4orf3 is required for UCP1-independent thermogenesis *in vivo* (A) Cold tolerance test of male *C4orf3*^CRISPRi^ mice and littermate controls. Mice kept at room temperature were exposed to cold (6°C) for indicated time points. *n* = 11. Statistic: two-way ANOVA with Šídák’s multiple comparisons test. (B) OCR in iBAT and IngWAT of male *C4orf3*^CRISPRi^ mice and control male mice following cold exposure. A subset of isolated tissues was stimulated with NE. *n* = 12 per group for iBAT; 10 per group for Ing WAT. Statistic: Mann-Whitney U test. (C) Relative mRNA expression of thermogenic genes in IngWAT of male mice following cold exposure. *n* = 5. (D) Mitochondrial respiration in IngWAT of male mice following cold exposure. *J*O_2_ at indicated states was measured following substrate injection. *n* = 5. (E) Heat rate in isolated mitochondria (state 4, complexes I and II) from IngWAT of male mice following cold exposure. Data are normalized by mitochondrial protein contents. *n* = 4. (F) Cold tolerance test of male UCP1 KO mice and DKO mice (UCP1 KO × *C4orf3*^CRISPRi^). The mice were chronically treated with the β3-adrenergic receptor agonist CL316,243 for 5 days to stimulate beige fat biogenesis in both groups. *n* = 8 per group. Statistic: two-way ANOVA with Šídák's multiple comparisons test and individual unpaired t tests. (G) Ca^2+^ uptake in isolated microsomes from IngWAT of male DKO mice and control UCP1 KO mice at pCa^2+^ 6.0. *n* = 4. (H) SERCA ATP hydrolysis assay in isolated microsomes from (G). The values were normalized by microsomal protein contents. (I) The bioenergetic efficiency of SERCA2 was calculated by Ca^2+^ uptake per ATP hydrolysis from (G) and (H). (J) Heat rate in isolated microsomes from IngWAT of UCP1 KO and DKO male mice at pCa 6.0. Thapsigargin was added to calculate SERCA-dependent thermogenesis. *n* = 4 per group. Statistic (C–E and G–J): unpaired t test. Bars represent the mean and error shown as SEM.

We aimed to determine the functional requirement of C4orf3/ALN for adipose tissue thermogenesis and energy balance *in vivo*. To this end, we exposed CRISPRi-*C4orf3* mice and littermate controls to 6°C. However, we did not find any difference in their rectal temperature during cold exposure (Figure 4A). Since cold-induced muscle shivering was equally active in both groups (Figure S4A), we next examined possible compensatory non-shivering thermogenic responses in adipose tissues. We found that OCR in the iBAT of CRISPRi-*C4orf3* mice was significantly higher than that of controls when the tissues were stimulated with NE. Similarly, the OCR of the inguinal WAT of CRISPRi-*C4orf3* mice was higher than that of controls under basal and NE-stimulated conditions (Figure 4B). The elevated OCR in the inguinal WAT was accompanied by increased mRNA expression of thermogenic genes, including *Ucp1*, *Cidea*, *Elov3*, and *Dio2* (Figure 4C). There was a trend of elevated *Ucp1* and *Dio2* mRNA expression in the iBAT of CRISPRi-*C4orf3* mice relative to that of control mice, although there was no difference in UCP1 protein content in the iBAT between the genotypes (Figures S4B and S4C).

The above results led to the hypothesis that cold exposure triggered a compensatory activation of UCP1-mediated thermogenesis in CRISPRi-*C4orf3* mice. To test this, we isolated mitochondria from the inguinal WAT and the iBAT of cold-exposed mice. In the inguinal WAT, we found that OCRs related to mitochondrial complexes I and II activities of C4orf3/ALN-deficient mice were significantly higher than those of controls (Figure 4D). Furthermore, protein expression levels of mitochondrial subunits in complexes II (SDHB), III (UQCRCII), IV (MTCO1), and V (ATP5A) were higher in the inguinal WAT of C4orf3/ALN-deficient mice than in control mice (Figure S4D). By contrast, there was no difference in iBAT mitochondrial respiration and the complex subunit expression between the two groups (Figures S4E and S4F). To directly examine changes in heat production in these tissues, we then performed ITC-based thermogenesis assays in the isolated mitochondria of cold-exposed mice. We found that mitochondrial thermogenesis in the inguinal WAT of CRISPRi-*C4orf3* mice was significantly higher relative to control mice (Figure 4E). There was a trend of increase in TNAP protein levels in the inguinal WAT of CRISPRi-*C4orf3* mice; however, pharmacological inhibition of creatine cycling using SBI-425, a TNAP inhibitor, did not alter mitochondrial respiration of CRISPRi-*C4orf3* mice (Figure S4G). These results indicate that UCP1-dependent thermogenesis mediates the compensatory activation of mitochondrial thermogenesis in CRISPRi-*C4orf3* mice.

Thus, we generated mice lacking both UCP1 and C4orf3/ALN (DKO mice) by breeding CRISPRi-*C4orf3* mice with *UCP1 KO* mice. Subsequently, the mice were chronically treated with the β3-adrenergic receptor agonist CL316,243 for 5 days to stimulate beige fat biogenesis in both groups. These mice were kept at ambient temperature and then exposed to 6°C. In alignment with a previous work showing WAT thermogenesis in UCP1 KO mice,^9^^,^^10^ CL316,243-treated *UCP1* KO mice were able to maintain their core body temperature for up to 6 h following cold exposure (Figure 4F). However, the rectal temperature of DKO mice rapidly decreased and reached below 30°C at 5 h. At 6 h after cold exposure, DKO mice developed hypothermia, and thus, we terminated the experiment to avoid hypothermia-induced death. To further determine the UCP1-independent function of C4orf3/ALN in the inguinal WAT of DKO mice, we then harvested the microsomes in which we performed Ca^2+^ uptake assays at pCa 6.0. Consistent with the results in CRISPRi-*C4orf3* mice, we found that DKO mice displayed significantly higher Ca^2+^ uptake in the microsomes than control *Ucp1* KO mice (Figure 4G), while they showed slightly lower ATPase activity than controls (Figure 4H). Accordingly, the inguinal WAT microsomes of DKO mice displayed higher SERCA2 efficiency (Ca^2+^ uptake/ATP hydrolysis) than that of control *Ucp1* KO mice (Figure 4I). Importantly, the ITC assay revealed a significant decrease in SERCA-dependent microsomal thermogenesis in DKO mice compared with control *Ucp1* KO mice (Figure 4J). These results indicate that C4orf3/ALN is required for UCP1-independent thermogenesis and cold adaptation *in vivo*.

### Compensatory responses to C4orf3 and UCP1 loss

**Figure 5:**
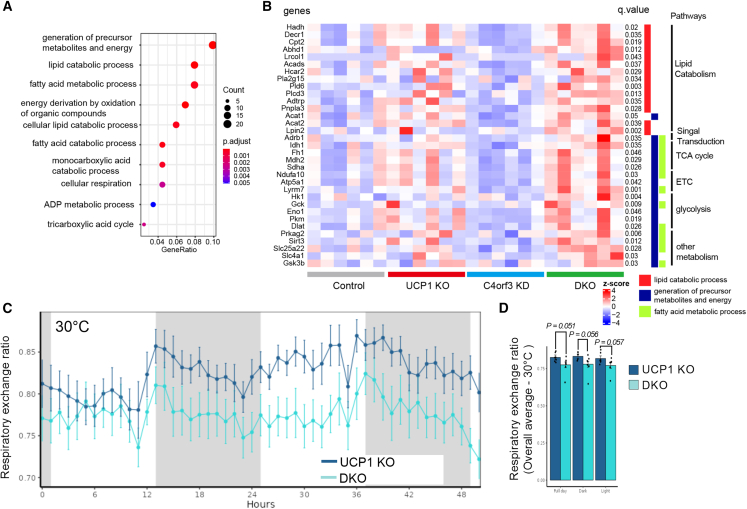
Compensatory responses to C4orf3 and UCP1 loss (A) GO pathway enrichment analysis for 276 genes that were uniquely upregulated in IngWAT of DKO mice (UCP1 KO × *C4orf3*^CRISPRi^) relative to other genotypes following cold exposure. *n* = 6 per group. (B) Relative mRNA levels of indicated genes in IngWAT of WT control, UCP1 KO, *C4orf3*^CRISPRi^, and DKO mice (UCP1 KO × *C4orf3*^CRISPRi^) following cold exposure at 6°C for 5 h. Data represented as *Z* score heatmap for each gene in each sample representing quantitated value. GO terms and pathways for each gene are listed on the right. *n* = 6. (C) The respiratory exchange ratio of mice at 30°C. *n* = 7 per group. (D) Quantification of respiratory exchange ratio in (C). Statistic: unpaired t test.

To better understand molecular changes associated with C4orf3/ALN and UCP1 loss, we next performed RNA-seq analyses in the inguinal WAT of DKO mice following cold exposure for 5 h. The RNA-seq analysis led to the following observations. First, UCP1 loss resulted in elevated expression of genes involved in Ca^2+^ cycling thermogenesis, including *Atp2a2* (SERCA2), *Ryr2*, and *Itpr1* (Figure S5A). This is consistent with our previous study.^7^ Similarly, the expression of creatine cycling genes *Slc6a8* and *Ckm* was elevated in the absence of UCP1 (Figure S5B). Second, we identified 276 genes that were preferentially upregulated in mice lacking both C4orf3/ALN and UCP1 compared with other genotypes (Table S1). Gene Ontology (GO) analysis of this gene set indicated the upregulated pathways were related to (1) the generation of precursor metabolites and energy, (2) lipid catabolic process, and (3) fatty acid metabolic process (Figure 5A). The gene programs include β1-adrenergic receptor (*Adrb1*), carnitine palmitoyltransferase II (*Cpt2*), adiponutrin (*Pnpla3*), phospholipase C delta 3 (*Plcd3*), acetyl-coenzyme A (CoA) acetyltransferase1/2 (*Acat1* and *Acat2*), *Sirt3*, and *Prkag2* (AMPK subunit γ2) (Figure 5B). Of note, the respiratory exchange ratio of DKO was lower than that of *Ucp1* KO mice (Figures 5C and 5D), supporting the transcriptome data that lipid catabolic processes and fatty acid oxidation are augmented in the inguinal WAT as a compensatory pathway of cold adaptation when both Ca^2+^ cycling and UCP1 thermogenesis are inactivated.

### Loss of C4orf3 resulted in increased adiposity and systemic insulin resistance

**Figure 6:**
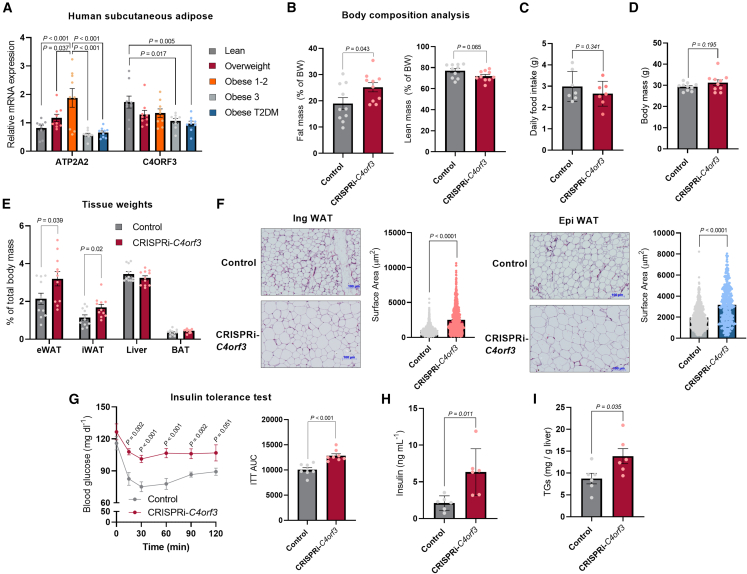
Loss of C4orf3 resulted in increased adiposity and systemic insulin resistance (A) Relative mRNA expression of indicated genes in the subcutaneous adipose tissue of people in Table 1. Lean: *n* = 6 female, *n* = 3 males; overweight: *n* = 5 female, *n* = 4 males; obese classes 1 and 2: *n* = 6 female, *n* = 3 males; obese class 3: *n* = 9 female, *n* = 1 male; obese with T2D: *n* = 5 female, *n* = 4 males. Statistic: one-way ANOVA with Tukey’s post hoc HSD test. (B) Fat mass and lean mass of male *C4orf3*^CRISPRi^ and littermate control mice at 30°C on a regular chow diet. *n* = 10. (C) Daily food intake of mice in (B). (D) Body weight of mice in (B) at 20 weeks old. (E) Indicated tissue weight of male mice on a regular diet at 30°C. *n* = 11. (F) H&E staining and adipocyte size of IngWAT (*n* = 917 cells for control, 623 cells for *C4orf3*^CRISPRi^) and epididymal WAT (n = 699 cells for control, 364 for *C4orf3*^CRISPRi^). Male mice at 30°C on a regular chow diet (*n* = 3 per group). Statistic: unpaired t test with Welch’s correction. (G) Insulin tolerance test of male *C4orf3*^CRISPRi^ mice and controls after 4 h fasting (0.5 U per kg^−1^). *n* = 8. (H) Fasting insulin levels in male *C4orf3*^CRISPRi^ mice and control mice. *n* = 6. (I) Blood triglyceride (TG) levels in male *C4orf3*^CRISPRi^ mice and littermate control mice. *n* = 6. Statistic (C–E and G–I): unpaired t test. Bars represent the mean and error shown as SEM.

Given the high expression of C4ORF3 in adipose tissues of mice and humans, we next examined the expression of *C4ORF3* in human subcutaneous adipose tissues from people classified as being normal weight, overweight, and obese. Patient demographics are provided in Table 1. We found that the mRNA expression of *C4ORF3* was significantly lower in people with grade 3 obesity (BMI > 40) and obese individuals with type 2 diabetes relative to non-obese healthy people (Figure 6A). On the other hand, the mRNA expression of *ATP2A2* (SERCA2) was slightly elevated in people with grade 1 and 2 obesity but downregulated in people with grade 3 obesity and obesity with type 2 diabetes. Of note, human genetic association studies (genome-wide association study [GWAS]) from the Type 2 Diabetes Knowledge Portal (http://type2diabetesgenetics.org/) found modest yet significant associations between *C4ORF3* and BMI (*p* = 4.54e^−8^), LDL cholesterol (*p* = 2.14e^−9^), and coronary artery disease (*p* = 2.80e^−10^). These results indicate a possible role of C4orf3/ALN in systemic energy balance.

**Table 1:** Patient demographics

Group	Age (years)	Female	Male	BMI (kg/m^2^)	Fat %	Systolic BP	Diastolic BP
Normal weight	55.9 ± 4.5	6	3	23.1 ± 0.6	28 ± 2.4	129 ± 5.5	79.4 ± 3.3
Overweight	56.8 ± 4.4	5	4	27.5 ± 0.4	33.9 ± 2	140.9 ± 6.7	81.6 ± 3.9
Obese class 1 and 2	53 ± 3.4	6	3	34.4 ± 0.8	39 ± 1.9	137.7 ± 5.8	87.1 ± 1.6
Obese class 3	53.9 ± 4.1	9	1	46.2 ± 2.1	45.7 ± 1.2	135.6 ± 4.8	80.9 ± 3.3
Obese T2DM	58.1 ± 2	5	4	43.3 ± 2.7	44.7 ± 0.7	136.1 ± 4.5	80.8 ± 4.2

Age, body mass index (BMI), fat %, systolic blood pressure (BP), and diastolic BP are presented as mean ± SEM.

Hence, we tested the degree to which C4orf3/ALN deficiency alters systemic energy balance and glucose homeostasis. To minimize the contribution of UCP1 to cold and diet-induced thermogenesis, the experiments were conducted under a thermoneutral condition (30°C) on a regular chow diet. At 20 weeks of age, echo-MRI scans found that CRISPRi-*C4orf3* mice exhibited significantly higher adiposity than littermate controls and also a trend of reduced lean mass, although there were no significant differences in muscle weight, grip strength, and muscle fiber-type composition between the genotypes (Figures 6B and S6A–S6C). There was no statistically significant difference in whole-body OCR between the two groups at 30°C (Figures S6D and S6E). We also found no difference in food intake between the genotypes (Figure 6C). Accordingly, the overall body weight was not significantly different between the two groups (Figure 6D). Nonetheless, CRISPRi-*C4orf3* mice had significantly higher tissue mass of epididymal WAT and inguinal WAT than control mice (Figure 6E). The histological analyses showed that adipocyte size in the inguinal WAT and epididymal WAT of CRISPRi-*C4orf3* mice was significantly larger than that of control WAT depots (Figure 6F). Elevated adiposity was consistently found in female CRISPRi-*C4orf3* mice (Figure S6F).

Lastly, we asked if C4orf3/ALN loss affects systemic insulin sensitivity in these mice as a result of the increase in adiposity.^43^^,^^44^ Insulin tolerance tests showed that CRISPRi-*C4orf3* mice were significantly less insulin tolerant than control mice even on a regular chow diet (Figure 6G). Moreover, fasting insulin levels were also significantly higher in CRISPRi-*C4orf3* mice than in control mice (Figure 6H). We noted that triglyceride content was significantly elevated in the liver of CRISPRi-*C4orf3* mice, implicating its involvement in insulin resistance (Figure 6I). Consistently, female CRISPRi-*C4orf3* mice exhibited systemic insulin resistance relative to littermate control mice (Figure S6G). Together, these results suggest that C4orf3/ALN deficiency results in higher adiposity and systemic insulin resistance *in vivo*.

## Discussion

Previous work by us and others has attempted to assess the role of adipose tissue Ca^2+^ cycling in whole-body energy balance by characterizing fat-specific SERCA2 KO mice.^7^^,^^45^ Although fat-specific SERCA2 KO mice exhibited a defect in inguinal WAT thermogenesis and systemic insulin resistance,^7^^,^^45^ a caveat is that SERCA2 deletion abolishes intracellular Ca^2+^ flux and causes numerous defects beyond thermogenesis, including impaired adipokine secretion and increased ER stress. Thus, instead of deleting SERCA2, we searched for the molecular resistor to Ca^2+^ cycling. The present work shows that depletion of *C4orf3* does not abolish intracellular Ca^2+^ flux, but rather, it alters the stoichiometry of SERCA2-dependent ATP hydrolysis and Ca^2+^ import, i.e., the Ca^2+^ transport efficiency of SERCA2. Accordingly, the SERCA2-C4orf3 complex requires almost twice as much ATP to transport the same amount of Ca^2+^ as SERCA2 alone, rendering the SERCA2b-C4orf3 complex exothermic. Importantly, the identification of C4orf3/ALN enables us, for the first time, to rigorously address the extent to which Ca^2+^ cycling thermogenesis contributes to whole-body energy balance. With the use of ITC-based measurement of heat production at the organelle resolution, we also determined the contribution of microsomal Ca^2+^ cycling thermogenesis in adipose tissue. Our study showed that BAT thermogenesis via UCP1 is the dominant source relative to microsomes in the inguinal WAT, as UCP1 could compensate for impaired microsomal Ca^2+^ cycling thermogenesis in mice lacking *C4orf3*. Nevertheless, Ca^2+^ cycling thermogenesis plays a significant role in cold adaptation and energy balance when UCP1 is inactive (e.g., *Ucp1* loss or chronic thermoneutrality). These results indicate that Ca^2+^ cycling thermogenesis in adipose tissue is a compensatory pathway for UCP1-dependent mitochondrial thermogenesis.

From structural and biophysical points of view, the SERCA2b-C4orf3 complex can be interpreted as a unique thermogenic machinery. First, among all the SERCA isoforms (SERCA1, SERCA2a, 2b, SERCA3), SERCA2b displays the highest Ca^2+^ affinity due to its unique C-terminal extension (11^th^ TM helix and the subsequent 12-residue tail) and a nearly 10-fold lower rate of Ca^2+^ dissociation from the E1⋅ Ca^2+^ state compared with SERCA2a.^46^^,^^47^ These properties are suitable for efficiently capturing Ca^2+^ but also inefficiently releasing Ca^2+^ into the ER lumen side. Second, C4orf3/ALN (65 amino acids) has an unusually long cytosolic domain compared with other known SERCA-binding peptides, such as SLN (31 amino acids). SLN resides within the SR membrane and interacts with SERCA1 exclusively at the intramembrane groove surrounded by TM2, TM4, TM6, and TM9.^39^^,^^40^^,^^41^ The constitutive binding of SLN to SERCA1 likely results in an inhibitory conformation and dynamics of this Ca^2+^-ATPase.^23^^,^^46^ By contrast, the cytosolic domain of C4orf3 interacts with the P-domain, which contains a catalytic site essential for ATP hydrolysis.^48^^,^^49^ The interaction between C4orf3 and SERCA2b reduces the energetic efficiency of SERCA2-dependent Ca^2+^ transport into the ER lumen while its ATPase activity is rather enhanced. This unique interaction enables the exothermic property of the SERCA2b-C4orf3 complex by dissipating ATP-derived energy in the form of heat. Detailed structural analyses of the SERCA2b-C4orf3 complex at the E1 and E2 states will further elucidate how C4orf3 binding modulates the ATP-driven Ca^2+^ transport efficiency of SERA2b.

A notable observation in this study is that UCP1 and UCP1-independent thermogenic pathways are regulated in a coordinated manner to maintain overall heat production and energy balance. For instance, Ca^2+^ cycling thermogenesis was activated in UCP1 KO mice, whereas C4orf3 loss resulted in the augmentation of UCP1-dependent thermogenesis in the mitochondria. When both Ca^2+^ cycling and UCP1 were inactivated, lipid catabolic processes, possibly related to futile lipid cycling, were enhanced in inguinal WAT. Intriguingly, such compensatory regulation has been reported in the context of creatine cycling, where UCP1 expression was upregulated in the BAT of fat-specific CKB KO mice following cold exposure.^50^ Additionally, cellular respiration linked to lipid cycling was augmented in cultured UCP1-null brown adipocytes.^18^ It is also notable that recent single-cell analyses of mouse and human adipose tissues identified distinct populations of adipocytes that either utilize UCP1 or futile creatine cycling.^31^^,^^51^ Considering the highly heterogeneous nature of adipose tissue, understanding the compensatory mechanisms of UCP1 vs. UCP1-independent pathways at the single-cell resolution is an important area for future research.

### Limitations of the study

We are aware that C4orf3/ALN is expressed in multiple tissues, including the liver, heart, and brain; however, our study found that mice lacking *C4orf3* exhibited reduced microsomal thermogenesis selectively to the inguinal WAT. The molecular basis for such tissue selectivity awaits future studies. We are also aware that a decrease in systemic insulin sensitivity may not be mediated by reduced thermogenesis in adipose tissue alone. Although C4orf3 expression is nearly undetected in the skeletal muscle, the current data do not rule out the possibility that the reduced lean mass contributes to insulin resistance in CRISPRi-*C4orf3* mice. Lastly, the quantitative contributions of Ca^2+^ cycling relative to other UCP1-independent pathways to the overall energy balance will be an important future topic.

## Resource availability

### Lead contact

Further information and requests for resources and reagents should be directed to and will be fulfilled by the lead contact, Shingo Kajimura (skajimur@bidmc.harvard.edu).

### Materials availability

Mouse strains and plasmids generated in this study are available upon request from the lead contact.

### Data and code availability

• The raw and processed data generated in this study have been deposited in the Gene Expression Omnibus (GEO) database under accession code GEO: GSE268770. The source data are provided as a Source Data file.
• The mass spectrometry proteomics data have been deposited to the ProteomeXchange Consortium via the PRIDE partner repository with the dataset identifier PRIDE: PXD059431.
• Source data, full-trace ITC data, and uncropped western blots are available in Data S1.
• This paper does not report original code.
• Any additional information required to reanalyze the data reported in this work paper is available from the lead contact upon request.

## Acknowledgments

We are grateful to T. Yamamuro, D. Kato, Y. Higuchi, M. Granath-Panelo, and Z. Zhao in the Kajimura lab for their technical support. We also thank the Thermo Fisher Center for Multiplexed Proteomics at Harvard Medical School for the proteomics analysis. We thank the staff at the Edinburgh Clinical Research Facility for their assistance. This work was supported by grants from the National Institutes of Health (NIH) (DK097441 and DK126160) and the Howard Hughes Medical Institute to S.K. R.H.S. is supported by the Medical Research Council (MR/S035761/1 and MR/W01937X/1). K.I. is supported by AMED-CREST from the Japan Agency for Medical Research and Development (AMED) (24gm1410006h9904). This work is also supported by the Cooperative Research Project Program of the Medical Institute of Bioregulation, Kyushu University. A.R.P.V. is supported by King Trust, Bank of America Private Bank, Co-Trustees. M.F. is supported by a Suzuki Manpei Diabetes Foundation Fellowship and Kowa Life Science Foundation Fellowship. S.O. is supported by the Japan Society for the Promotion of Science (JSPS) and the Uehara Memorial Foundation.

## Author contributions

Conceptualization, C.A., K.S., and S.K.; methodology, A.R.P.V., C.A., J.-S.Y., K. Ikeda, M.L., and T.R.O.; investigation, A.R.P.V., C.A., C.X., J.-S.Y., K. Ikeda, K.S., M.F., M.L., M.P.H.C., S.O., and T.R.O.; validation, C.A., K. Ikeda, K. Inaba, and M.L.; formal analysis, C.A., K. Inaba, M.L., and R.H.S.; writing – original draft, S.K.; writing – review and editing, C.A., R.H.S., and S.K.; funding acquisition, K. Inaba, K.S., P.R.G., R.H.S., and S.K.; supervision, K. Inaba, P.R.G., R.H.S., and S.K.

## Declaration of interests

The authors declare no competing interests.

## STAR★Methods

### Key resources table

**Table:** 

REAGENT or RESOURCE	SOURCE	IDENTIFIER
**Antibodies**		

ATP2A2	Thermo Fisher Scientific	Cat# MA3-919; RRID:AB_325502
Calreticulin	Cell Signaling	Cat# 12238; RRID:AB_2688013
FLAG	Sigma-Aldrich	Cat# F1804; RRID: AB_262044
β-actin	Cell Signaling	Cat# 8457; RRID:AB_10950489
C4ORF3	This paper	N/A
Anti-DYKDDDDK magnetic beads	Sigma-Aldrich	Cat# M8823; RRID:AB_2637089
Streptavidin magnetic beads	Thermo Fisher Scientific	Cat# 65001
Total oxphos cocktail	Abcam	Cat# ab110413; RRID: AB_2629281
TNAP	R&D Systems	Cat# AF2910-SP
COX IV	Cell Signaling	Cat# 38563; RRID:AB_2799136
UCP1	Abcam	Cat# ab10983; RRID:AB_2241462
Goat anti-mouse IgG (H&L) secondary antibody, HRP	Thermo Fisher Scientific	Cat# 31430; RRID: AB_10960845
Goat anti-rabbit IgG (H&L) secondary antibody, HRP	Abcam	Cat# ab6721; RRID: AB_955447

**Biological samples**		

Human adipose tissue	This paper	N/A

**Chemicals, peptides, and recombinant proteins**		

Indomethacin	Sigma-Aldrich	Cat# I7378
Insulin	Sigma-Aldrich	Cat# I6634
Adenylyl imidodiphosphate	MedChemExpress	Cat# HY-130777
Isobutylmethylxanthine (IBMX)	Sigma-Aldrich	Cat# I5879
Dexamethasone	Sigma-Aldrich	Cat# D4902
3,3′,5-triiodo-L-thyronine (T_3_)	Sigma-Aldrich	Cat# T2877
L-(-)-Norepinephrine(+)-bitartrate salt monohydrate	Sigma-Aldrich	Cat# A9512
DMEM	Gibco	Cat# 11965092
Fetal Bovine Serum	ATLANTA biologicals	Cat# S11550
Penicillin-Streptomycin	Gibco	Cat# 15140
Basticidin S HCl (10 mg/mL)	Gibco	Cat# A1113903
0.05% Trypsin	Corning	Cat# MT25052CI
cOmplete, EDTA-free Protease Inhibitor Cocktail	Roche	Cat# 11873580001
Phosphatase inhibitor cocktail 2	Sigma-Aldrich	Cat# P5726
Phosphatase inhibitor cocktail 3	Sigma-Aldrich	Cat# P0044
Dextrose	Sigma-Aldrich	Cat# D9434
Palmitoyl-L-carnitine	Sigma-Aldrich	Cat# 61251
Bovine Serum Albumin	Sigma-Aldrich	Cat# A1595
Malate	Sigma-Aldrich	Cat# M1000
Succinate	Sigma-Aldrich	Cat# 224731
Glutamate	Sigma-Aldrich	Cat# G1501
Rotenone	Sigma-Aldrich	Cat# R8875
N,N.N’,N’-Tetramethyl-p-phenylenediamine	Sigma-Aldrich	Cat# T7394
L-ascorbate	Sigma-Aldrich	Cat# A4034
Thapsigargin	Sigma-Aldrich	Cat# T9033
^45^CaCl_2_	PerkinElmer	Cat# NEZ013001MC
CaCl_2_	Sigma-Aldrich	Cat# C3306
QIAzol lysis reagent	Qiagen	Cat# 79306
TRIzol reagent	Thermo Fisher Scientific	Cat# 15596026
Adenosine 5’-diphosphate	Sigma-Aldrich	Cat# A5285
MgCl_2_	Sigma-Aldrich	Cat# M2393
NaCl	Sigma-Aldrich	Cat# S7653
KCl	Sigma-Aldrich	Cat# P9541
Na_2_HPO_4_	Fluka	Cat# 71639
NaH_2_PO_4_	Sigma-Aldrich	Cat# 71505
Adenosine 5’-triphosphate	Sigma-Aldrich	Cat# A2383
Sucrose	Sigma-Aldrich	Cat# S0389
D-mannitol	Sigma-Aldrich	Cat# M4125
Scintillation fluid	PerkinElmer	Cat# 6013326
Tris	Sigma-Aldrich	Cat# 11814273001
EGTA	Sigma-Aldrich	Cat# E4378
n-Dodecyl β-D-maltoside	Sigma-Aldrich	Cat# D4641
DTT	Sigma-Aldrich	Cat# 43816
Glycerol	Sigma-Aldrich	Cat# G7793
3X-FLAG peptide	Sigma-Aldrich	Cat# F4799
Paraformaldehyde	Santa Cruz Biotechnology	Cat# SC281692
PBS	Gibco	Cat# 10010023
NaN_3_	Sigma-Aldrich	Cat# S2002
MES potassium salt	Santa Cruz	Cat# sc-286156
Chloroform	Sigma-Aldrich	Cat# 650498
Acetonitrile	Thermo Fisher Scientific	Cat# A955
Methanol	Thermo Fisher Scientific	Cat# A456
Rosiglitazone	Enzo Life Sciences	Cat# ALX-350-125
Hygromycin B	Thermo Fisher Scientific	Cat# 10687010
2,2,2-Tribromoethanol	Sigma-Aldrich	Cat# T48402
KH_2_PO_4_	Sigma-Aldrich	Cat# P5655
GlutaMAX supplement	Thermo Fisher Scientific	Cat# 30055061
Antimycin A	Sigma-Aldrich	Cat# A8674
Water LC/MS	Thermo Fisher Scientific	Cat# W64
Formic acid	Thermo Fisher Scientific	Cat# A117
Guanosine 5’-diphosphate	Sigma-Aldrich	Cat# G7127
SDS	Thermo Fisher Scientific	Cat# BP166
HEPES	Sigma-Aldrich	Cat# H3375
LiCl	Sigma-Aldrich	Cat# L4408
EDTA	Sigma-Aldrich	Cat# E5134
NP-40	Boston Bioproducts	Cat# P-877
Sodium deoxycholate	Sigma-Aldrich	Cat# D6750
Triton-X 100	Thermo Fisher Scientific	Cat# BP151
Tween 20	Thermo Fisher Scientific	Cat# BP337
KOH	Boston Bioproducts	Cat# BZ-8038
Rac-Glycerol 1-Phosphate	Santa Cruz	Cat# sc-215789
Pyruvate	Sigma-Aldrich	Cat# P4562
HBSS	Thermo Fisher Scientific	Cat# 88284
XF calibrant solution	Agilent	Cat# 100840-000
SBI-425	MedChemExpress	Cat# HY-124756
Creatine	Sigma-Aldrich	Cat# C3630
Laemmli sample buffer	Bio-rad	Cat# 1610747
β-mercaptoethanol	Sigma-Aldrich	Cat# M3148
Potassium oxalate	Sigma-Aldrich	Cat# P0963
MOPS	Sigma-Aldrich	Cat# M3183
Lauryl maltose neopentyl glycol	Thermo Fisher Scientific	Cat# A50940
Biotin	Sigma-Aldrich	Cat# B4639
Urea	Sigma-Aldrich	Cat# 51456
Bismaleimidohexane	Thermo Fisher Scientific	Cat# 22330
Polybrene transfection reagent	Sigma-Aldrich	Cat# TR-1003-G
KF	Sigma-Aldrich	Cat# 449148
AlCl_3_	Sigma-Aldrich	Cat# 237051
CL 316,243	Sigma-Aldrich	Cat# C5976
Phenylmethanesulfonyl fluoride	Sigma-Aldrich	Cat# 93482
DNAse	Qiagen	Cat# 79254
ERCC RNA spike-in mix	Thermo Fisher Scientific	Cat# 4456740

**Critical commercial assays**		

RNeasy lipid tissue mini kit	Qiagen	Cat# 74804
Fluo-8 calcium flux assay kit	Abcam	Cat# ab112129
EnzChek phosphate assay kit	Thermo Fisher Scientific	Cat# E6646
Triglyceride quantification kit	Abcam	Cat# ab65336
iScript Reverse Transcription Supermix for rt-qPCR	Bio-rad	Cat# 1708841
iTaq Universal SYBR Green Supermix	Bio-rad	Cat# 1725125
Biorad gels 4-20% 15 well	Bio-rad	Cat# 4561096
Seahorse XF24 islet capture Fluxpak	Agilent	Cat# 101122-100
Pierce BCA Protein Assay Kit	Thermo Fisher Scientific	Cat# 23225
Rat/mouse insulin ELISA	Sigma-Aldrich	Cat# EZRMI
Seahorse XFe24 FluxPak	Agilent	Cat# 102340-100
Glucometer	Abbott	Cat# Freestyle Lite
Glucose strips	Abbott	Cat# 70827
Biorad gels 12% 10 well	Bio-rad	Cat# 4568044
RNeasy mini kit	Qiagen	Cat# 74104
FastSelect –rRNA HMR Kits	Qiagen	Cat# 334386
NEBNext Ultra II RNA Library Prep Kit for Illumina	New England Biolabs	Cat# E7770L

**Deposited data**		

RNA sequencing	This paper	GSE268770
Proteomics	This paper	PXD059431

**Experimental models: Cell lines**		

ALN Mutant_A overexpressing 293T	This paper	N/A
ALN Mutant_B overexpressing 293T	This paper	N/A
ALN Mutant_C overexpressing 293T	This paper	N/A
ALN Mutant_TM overexpressing 293T	This paper	N/A
ALN wild type overexpressing 293T	This paper	N/A
*C4orf3*-TurboID immortalized inguinal SVF	This paper	N/A
CRISPRi-*C4orf3* immortalized inguinal SVF	This paper	N/A
W48C ALN mutant-overexpressing inguinal SVF	This paper	N/A
Immortalized inguinal SVF	Kajimura Lab^43^	N/A
SERCA2b-overxpressing inguinal adipocytes	This paper	N/A

**Experimental models: Organisms/strains**		

Mouse: C57BL6J mice	Jackson Laboratory	Cat# 000664
Mouse: CRISPRi-*C4orf3*	This paper	N/A
Mouse: dCas9/KRAB	Kajimura lab^51^	N/A
Mouse: UCP1 KO	Jackson Laboratory	Cat# 003124
Mouse: UCP1 x C4orf3 double knockout	This paper	N/A

**Oligonucleotides**		

A full list of qPCR primers in Table S2	This paper	N/A

**Recombinant DNA**		

AAV8-CAG-EGFP-U6-Scramble	VectorBuilder	N/A
AAV8-CAG-EGFP-U6-gRNA-long tracr	VectorBuilder	N/A

**Software and algorithms**		

CaIR	Banks Lab	https://calrapp.org/
Biorender	Biorender	https://biorender.com/
Protter	Protter	https://wlab.ethz.ch/protter/start/
GO Enrichment Analysis	GeneOntology	http://geneontology.org/
GraphPad Prism 8	GraphPad Software	https://www.graphpad.com/scientific-software/prism/
ImageJ	ImageJ Software	https://imagej.nih.gov/ij/download.html
R	Open Source	https://www.r-project.org/
Trimmomatic v.0.36	Open Source	http://www.usadellab.org/cms/?page=trimmomatic
STAR aligner v2.5.2b	Open Source	https://github.com/alexdobin/STAR
Subread package v1.5.2	Open Source	https://subread.sourceforge.net/
edgeR	Open Source	https://bioconductor.org/packages/release/bioc/html/edgeR.html
clusterProfiler	Open Source	https://bioconductor.org/packages/release/bioc/html/clusterProfiler.html
Pheatmap	Open Source	https://www.rdocumentation.org/packages/pheatmap/versions/1.0.12/topics/pheatmap
HDOCK server	Open Source	http://hdock.phys.hust.edu.cn
AlphaFold server	Open Source	https://alphafoldserver.com

**Other**		

Standard Diet	Lab Diet	Cat# 5008

### Experimental model and study participant details

#### Mouse Strains and Husbandry

All procedures performed in this study were approved by the Institutional Animal Care and Use Committee (IACUC) at Beth Israel Deaconess Medical Center and University of California, San Francisco (UCSF). Mice were housed at 23°C or 30°C in ventilated cages under a 12 h – 12 h light/dark cycle. Mice were fed a standard diet (Lab Diet 5008) with free access to food and water unless specified. For the generation of mice lacking *C4orf3*/ALN, we developed CRISPRi-*C4orf3* mice following the methodology that was described previously.^52^ In brief, we first screened the effective gRNAs to deplete *C4orf3*/ALN using dCas9-KRAB-derived mouse embryonic fibroblasts (MEFs). The most effective gRNA (5’-TTGGCGGGGTTA CCCGGAAT-3’) was inserted into the H11 locus to generate gRNA^*C4orf3*^ transgenic mice following established protocols. Subsequently, gRNA^*C4orf3*^ transgenic mice were crossed with CRISPRi mice that expressed the nuclease-deficient Cas9 fused to the zinc-finger protein 10 (ZNF10) Krüppel-Associated Box (KRAB). Ing WAT-specific *C4orf3* KD mice were generated by injecting AAV expressing gRNA-*C4orf3* (AAV8-CAG-EGFP-U6-gRNA-long tracr; custom order, VectorBuilder) directly into Ing WAT, while control mice were injected with AAV8-CAG-EGFP-U6-Scramble. Thirty μl of AAV at a dose of 1 × 10^10^ genomic copies (GC) μl^−1^ was injected in each Ing WAT depot (10 μl per injection, 3 locations per depot) for a viral titer of 6.0 x 10^11^ GC per mouse. Efficacy of viral infection and knockdown was evaluated by quantification of *C4orf3* expression level. To develop *Ucp1* x *C4orf3* DKO mice (DKO mice), the above-described CRISPRi-*C4orf3* mice were crossed with *Ucp1* KO mice (Strain # 003124, The Jackson Laboratory). Mice were on a C57BL/6J background.

#### Human Participants

Human adipose tissue samples were obtained from individuals undergoing elective abdominal surgery in the Royal Infirmary of Edinburgh. Local ethical approval was obtained (research ethics committee number 15/ES/0094/ 20/ES/0061) as was consent from all participants. For abdominal surgery, abdominal subcutaneous (SAT) samples were obtained intraoperatively from individuals undergoing hernia, cholecystectomy, or bariatric surgery. Tissue samples were immediately frozen at -80°C for subsequent RT-qPCR analyses.

#### Cell Culture

The stromal vascular fractions (SVFs) from inguinal WAT of wild-type mice (C57BL/6J), CRISPRi-*C4orf3* mice, and control mice (dCas9-KRAB) were isolated by collagenase digestion per the established protocol.^53^ Inguinal WAT-derived SVFs were immortalized by expressing the SV40 large T antigen as described previously.^54^^,^^55^ Preadipocytes were seeded into coated plates, and differentiation was induced by culturing cells with DMEM medium containing 10% FBS and an adipogenic cocktail consisting of 5 μg/ml insulin, 1 nM T3, 0.5 μM rosiglitazone, 0.5 mM isobutylmethylxanthine, 125 nM indomethacin and 2 μg/ml dexamethasone. After 48 h, the cell medium was changed to one containing 10% FBS, 5 μg/ml insulin, 1 nM T3 and 0.5 μM rosiglitazone for another 5–7 days. HEK293T cells were maintained in DMEM containing high glucose, 10% FBS and 1% penicillin–streptomycin.

### Method details

#### DNA Constructs

The construct for the transmembrane domain of ALN (ALN-TM) was generated with gBlocks (Integrated DNA Technologies), which contained the Kozak sequence, HA-tag, GS-linker, and ALN-TM (5′-GCCACCATGTACCCATACGATGTTCCAGATTACGCTGGTGGCGGAGGG TCTGGTGGCGGAGGGTCCGGTGGCGGAGGGTCATCCTACTGGCTGGATCTCTGGCTCTTCATCCTTTTCGACCTGGCCTTGTTCGTCTTCGTGTACCTCTTGCCCTAA-3’). The synthesized construct was inserted into Empty pMSCV-blasticidin vector (75085; Addgene) that was digested with BglII and EcoRI. The synthesized ALN-TM construct with inserted using In-Fusion Snap Assembly (Takara Bio) and transformed in Dh5a competent cells (NEB). ALN mutants (TM, A, B and C) were also cloned into the pMSCV-blasticidin vector using gBlocks Gene Fragments designed to generate the desired mutant (Figure S3D). Constructs were confirmed by sequencing and retrovirus was produced in HEK293 packaging cells using 10 μg of plasmid and 5 μg each of the packaging plasmids VSV and gag-pol via the calcium phosphate transfection method. The viral supernatant was collected and filtered (0.45 μm) 48 h later and 293 cells were infected with virus and 10 μg/mL polybrene for 24 h. Blasticidin (10 μg/mL, A1113903; Thermo Fisher Scientific) was used for the selection of stable cells. For assays of SERCA2b-mediated thermogenesis in HEK293 cells, mouse *Atp2a2b* was cloned into a pcDNA3.1 backbone and transiently expressed using Lipofectamine 3000 reagent (ThermoFisher).

#### Isolation of Microsomes and Mitochondria

To isolate microsomes, harvested cells and murine tissue were homogenized in 250 mM sucrose, 150 mM KCl, 10 mM Tris-HCl (pH 7.5), 100 nM CaCl_2_ and 0.2% NaN_3_, then centrifuged at 10,000 x g for 20 min at 4°C. The supernatant was ultracentrifuged at 100,000 x g for 1h at 4°C. Microsomes were then resuspended in a minimal volume of 50 mM HEPES (pH 7.0), 100 mM NaCl and 20% glycerol. For mitochondrial isolation, mouse inguinal WAT and interscapular BAT depots were excised and washed with ice-cold MHSE buffer (70 mM sucrose, 210 mM mannitol, 5 mM HEPES, 1 mM EGTA, pH 7.2) then minced and homogenized with a motorized Teflon glass homogenizer in MHSE buffer with 0.5 % (w/v) fatty-acid free BSA. The homogenate was centrifuged at 700 x g for 10 min at 4°C. Following the removal of fat and lipid by careful aspiration, the supernatant was passed through two layers of cheesecloth and centrifuged at 9,000 x g for 10 min at 4°C to obtain a mitochondrial pellet. After resuspending the pellet in MHSE + BSA, the 9,000 x g centrifugation step was repeated, and the final pellet was resuspended in a minimal volume of MHSE. Protein concentration was determined using the bicinchoninic assay (Pierce).

#### Organelle Thermogenesis Assays

Heat production by microsomes or mitochondria was measured using an Affinity isothermal titration calorimeter (TA instruments). The reference cell was filled with ddH_2_O while the sample cell (350 μL) was filled with reaction medium. Twenty μg of mitochondria/microsomes in a volume of 40 μL were injected into the sample cell. For mitochondrial studies, reaction medium consisted of Buffer Z (1 mM EGTA, 5 mM MgCl_2_, 105 mM K-MES, 30 mM KCl, 10 mM KH_2_PO_4_, 5 mg/mL fatty acid-free BSA, pH 7.1). Complex I and II-driven state 4 respiration was probed by adding 10 mM glutamate, 2 mM malate, 5 mM pyruvate and 10 mM succinate to Buffer Z. State 4 respiration driven by β-oxidation was analyzed by adding 40 μM palmitoyl-carnitine and 1 mM malate. For SERCA-dependent thermogenesis, the reaction buffer consisted of 50 mM HEPES (pH 7.0), 100 mM NaCl, 20% glycerol, 50 mM NaN_3_, 5 mM MgCl_2_, 1 mM EGTA, free Ca^2+^ (pCa 6.0) and 1 mM ATP. As additional controls, we included samples without Ca^2+^ or in the presence of the non-hydrolyzable ATP analog AMP-PNP (1 mM) and Ca^2+^(pCa 6.0). The MAXCHELATOR program was used to calculate the desired concentration of Ca^2+^. A second reaction containing 15 μM thapsigargin, a non-competitive SERCA inhibitor, was performed and heat rates were subtracted from the first injection to determine the contribution of SERCA-dependent Ca^2+^ cycling. Following the protocol adopted by the previous report,^28^ heat production was determined for 7 min, while the heat change in the initial two minutes after injection was discarded to avoid artifacts such as heat resulting from non-specific binding of ions to the lipid membrane of mitochondria/microsomes or dilution in the reaction medium. No calcium ionophore was added to the reaction, as our initial optimization experiments did not see any changes in thermogenesis. To approximate the contribution of State 4 respiration and SERCA-mediated Ca^2+^ cycling to whole-tissue thermogenesis, the heat (in μW) from 20 μg of mitochondria or microsomes was averaged over a five-minute period and then scaled up to the total mitochondrial/microsomal protein content in inguinal WAT or iBAT.

#### Identification of SERCA-binding Peptides

To identify SERCA2b-binding peptides in adipocytes, we assembled a database of mRNA expressions for noncoding RNA in the inguinal WAT of UCP1 KO mice and PRDM16 x UCP1 KO mice.^7^ This was done by utilizing mouse genome mm10 coupled with annotations sourced from the NONCODE knowledgebase. Next, we performed *in silico* translation of ∼3,000 putative noncoding RNAs. For inclusion in this analysis, we selected the RNAs that exhibited high mRNA expression (TPM> 10) across all UCP1 KO samples (*n*=4). The Open Reading Frame Finder was employed to carry out the translations, with a cutoff set for sequences longer than 20 amino acids. Following the translations, we conducted a BLAST homology search (*E*-value < 0.1) to compare the sequences of the putative micropeptides, including known SERCA binding peptides, such as myoregulin, phospholamban, and sarcolipin. Subsequently, we generated 3D structural models of the micropeptides using I-TASSER and performed docking simulations with a SERCA1 3D structure. The HDOCK server was used for the docking simulations, with all parameters set to default values. The top two docking models, based on their docking scores, were selected for further analysis.

#### Oxygen Consumption Assays

To assess the oxygen consumption rate (OCR) of interscapular BAT and iWAT, adipose tissue biopsies (0.5 mg for BAT and 1.5 mg for iWAT) were harvested and secured in the wells of an XF24 Islet Capture Microplate. Following a 1 h pre-incubation in XF assay medium supplemented with 25 mM glucose, 1 mM sodium pyruvate, and 2 mM GlutaMAX (ThermoFisher Scientific), basal and subsequently norepinephrine (10 μM)-stimulated respiration was measured using a Seahorse XFe Extracellular Flux Analyzer (Agilent). Data were normalized to mg of tissue weight.

#### Mitochondrial Respiration (*J*O_2_) Measurement

*J*O_2_ in isolated mitochondria was measured using an Oroboros O2k respirometer (Oroboros Instruments, Austria) in Buffer Z (1 mM EGTA, 5 mM MgCl_2_, 105 mM K-MES, 30 mM KCl, 10 mM KH_2_PO_4_, 5 mg/mL fatty acid-free BSA, pH 7.1). For complex I-driven respiration, 10 mM glutamate, 2 mM malate and 5 mM pyruvate were added. ADP (4 mM) was used to stimulate state 3 respiration. Succinate (10 mM) was added to measure the activity of complex II. Rotenone (10 μM) was used to inhibit complex I. The glycerophosphate pathway was probed with 10 mM rac-glycerol 1-phosphate. Antimycin A (2.5 μM) was used to inhibit mitochondrial respiration by preventing the oxidation of ubiquinol to ubiquinone. To measure futile creatine cycling, Buffer Z was supplemented with 4% fatty acid-free BSA, 1 mM GDP, 10 mM pyruvate, and 5 mM malate. In the presence or absence of the TNAP inhibitor SBI-425 (0.01 mM), 100 μg of iWAT mitochondria were added, followed by creatine (0.01 mM) and ADP (0.1 mM).

#### Antibodies and Immunoblotting

We developed a rabbit polyclonal antibody targeting C4orf3/ALN by using three peptide sequences of mouse C4orf3 (RRGSFEAGRRNQDEC, EVSQAASGTDGVREC, and CAPQSGMNGLPKHSY) as antigens for immunization. The specificity of the signal was validated by immunoblotting in adipose tissues and adipocytes isolated from wild-type mice and CRISPRi-*C4orf3* mice. Secondary antibodies were anti-rabbit IgG (1:5000, ab6721; Abcam) and anti-mouse IgG (1:5000, 31430; Thermo Fisher). For immunoblotting, RIPA buffer was used to homogenize tissues following the addition of protease (Roche) and phosphatase (Sigma; p5726) inhibitors. Homogenates were centrifuged for 20 min at 16000 x g and 4°C to pellet debris, and supernatants were analyzed by BCA assay. Protein samples were heated (95°C) with Laemmli sample buffer containing 5% (v/v) β-mercaptoethanol and loaded in a 10, 12%, or 16% SDS-PAGE gel. Separated proteins were transferred to PVDF membranes using the Trans-Blot Turbo Transfer System (Bio-Rad). PVDF membrane blots were blocked in Tris-buffered saline (TBS) with 0.1% Tween-20 and 5% BSA for 1 h and incubated overnight at 4°C with rabbit anti-UCP1 (1:1000, ab10983; Abcam), anti-calreticulin (1:1000, 12238; Cell Signaling Technology), anti-TNAP (1:1000, MAB29092; R&D Sytems), anti-COX IV (1:1000, 38563; Cell Signaling Technology), anti-SERCA2 (1:1000, MA3-919; Invitrogen) or anti-β-actin (1:20,000, A385; Sigma-Aldrich). Mitochondrial complexes were detected using Total OXPHOS Rodent WB Antibody Cocktail (1:2,000, ab110413; Abcam).

#### Intracellular Ca^2+^ Uptake Assay

Primary adipocytes from control (dCas9-KRAB) and CRISPRi-*C4orf3* mice were differentiated on collagen-coated class bottom dishes according to the above-mentioned protocol. Intracellular Ca^2+^ levels were determined using the Fluo-8 No Wash Calcium Assay kit (ab112129; Abcam). Differentiated adipocytes were incubated with Fluo-8 in calcium-free Hanks’ balanced salt solution (HHBS) at 37°C for 30 min and subsequently incubated at room temperature for an additional 30 min. The buffer was then replaced by HHBS, including 1 mM CaCl_2_ for Ca^2+^ flux assays. Fluorescence was detected by the fluorescence filter Cube 2002 (ECHO Laboratories; Excitation 470/40 nm, dichroic 495 nm, emission 525/50 nm). Fluorescence intensity was monitored following addition of 1 μM norepinephrine. Image analyses were performed using ImageJ software (https://imagej.nih.gov/ij/download.html). Specifically, the circular region of interest (ROI) was placed onto differentiated adipocytes. Time-dependent changes in intracellular Ca^2+^ levels were recorded by quantifying the pixel intensity of the ROI and expressed as delta fluorescence ratio F/F0 or delta F/F0 relative to control, where F is the fluorescence intensity at a given time and F0 is the initial resting fluorescence intensity prior to stimuli. Background signal (signal from the area with no cells) was subtracted from all data.

#### SERCA ATP Hydrolysis Assay

Microsomal SERCA2b ATPase activity was measured in a buffer containing 50 mM HEPES (pH 7.0), 100 mM NaCl, 20% glycerol, 50 mM NaN_3_, 5mM MgCl_2_, and 1 mM EGTA with various physiological concentrations of free CaCl_2_ (per MAXCHELATOR). The time-dependent release of free phosphate was measured following the addition of 1 mM ATP using the EnzCheck phosphate assay kit (Thermo Fisher Scientific). Ten μg of microsomal protein was used for each reaction, and SERCA-dependent ATPase activity was determining by taking the difference of values with and without 15 μM thapsigargin, which inhibits the SERCA pump.

#### Microsome Ca^2+^ Uptake Assay

Microsome Ca^2+^ uptake was assessed using varying concentrations of ^45^Ca (NEZ013001MC; PerkinElmer) in a reaction mixture consisting of 40 mM imidazole (pH 7.0), 95 mM KCl, 5 mM NaN_3_, 5 mM MgCl_2_, 0.5 mM EGTA and 5 mM K^+^ oxalate. Reactions were initiated with 5 mM ATP and terminated by filtration through a 0.22 μm polyethersulfone membrane filter (GPWP04700; Millipore) rinsed three times with wash buffer (20 mM Tris-HCl (pH 7.0), 2 mM EGTA). Membranes were then processed for scintillation counting. The SERCA coupling efficiency was calculated by dividing the rate of Ca^2+^ uptake by the rate of SERCA ATPase activity.

#### Protein Purification of SERCA2b

A lentiviral mouse flag-tagged *Atp2a2b* (SERCA2b) ORF clone expression vector was obtained from GeneCopoaeia (Ex-Mm28155-Lv207) and introduced into inguinal SVF or HEK293 packaging cells with 10 μg of lentiviral vector, 7.5 μg of psPAX2 and 2.5 μg of pMD2.G using the calcium phosphate method. Stable inguinal SVF cells overexpressing SERCA2b were selected for using hygromycin B. Harvested cells were lysed using a Dounce homogenizer in a buffer containing 50 mM HEPES-NaOH (pH 7.0), 100 mM NaCl, 20% glycerol, 100 μM CaCl_2_, 1 mM MgCl_2_, 1 mM phenylmethylsulfonyl fluoride (PMSF), and protease inhibitor (Roche). To solubilize the membrane fraction, 1% (w/v) n-dodecyl β-D-maltoside was added followed by 30 min of gentle rotation at 4°C. The sample was centrifuged at 12 000 x g for 20 min at 4°C to remove insoluble material and the supernatant was incubated with Anti-FLAG M2 magnetic beads (M8823; Millipore) for 2 h at 4°C with gentle rotation. The beads were washed five times with buffer containing 50 mM HEPES-NaOH (pH 7.0), 100 mM NaCl, 20% glycerol, 100 μM CaCl_2_, 1 mM MgCl and 0.05% (w/v) lauryl maltose neopentyl glycol (LMNG). SERCA2b was eluted with 200 μg/mL of 3X FLAG peptide (F4799; Sigma Aldrich) in the same buffer. Co-immunoprecipitation of *C4orf3*/ALN was verified by immunoblotting as described above.

#### Turbo ID Proximity Protein Labeling

Turbo ID, a biotin ligase engineered for proximity labeling, was attached to the N-terminus of ALN in the pMSCV vector using the In-Fusion HD Cloning Plus kit (638909; Takara Bio). Fully differentiated beige adipocytes expressing empty vector or TurboID-ALN were used for the experiments. Cells were incubated with 50 μM biotin for 30 min, then washed and incubated in complete DMEM for 3 h. After this three-hour biotin washout period, cells were washed with ice-cold phosphate-buffered saline (PBS) and collected. Scraped cells were centrifuged (1500 rpm, 5 min, 4°C) and washed a second time with PBS. Following a second centrifugation, cells were resuspended in lysis buffer containing 1% Triton X-100, 130 mM NaCl, 2.5 mM MgCl_2_, 2 mM EGTA, 25 mM HEPES pH 7.4, and protease inhibitor (Roche). Samples were rotated for 30 min at 4°C to ensure lysis then centrifuged at 1300 rpm for 10 min. Supernatants were normalized to the same protein concentration using a BCA assay then incubated overnight with streptavidin beads (Dynabeads MyOne Streptavidin C1, 65001; Invitrogen), and then washed for 5 min each in the following buffers: 2x (2% SDS), 1x (50 mM HEPES pH 7.4, 500 mM NaCl, 1 mM EDTA, 0.1% deoxycholate, 1% Triton-X 100), 1x (10 mM Tris pH 7.4, 250 mM LiCl, 1 mM EDTA 0.5% NP-40, 0.5% deoxycholate), 2x (50 mM Tris pH 7.4, 50 mM NaCl). After the final wash, samples were resuspended (50 mM Tris pH 7.4, 50 mM NaCl), snap frozen, and stored at -80°C until on-bead digestion. Beads were then washed with 50 mM Tris (pH 8.5) buffer, followed by resuspension in 1 M urea, 50 mM tris (pH 8) and digested with 3 μg trypsin (Promega) at 37°C for 6 h. Beads were further washed three times with 1 M urea, 50 mM tris (pH 8.5). Acidified digested peptides were desalted using a Sep-pak. Peptides were then eluted with 70% acetonitrile, 0.1% TFA and dried in a speedvac. Dried samples were reconstituted with 200 mM EPPS buffer, pH 8.5, and labelled with TMTPro reagents (Thermo Fisher Scientific). Following incubation at room temperature for 2 h, the reactions were quenched with hydroxylamine to a final concentration of 0.5% (v/v). Samples were combined, further desalted over StageTips, finally eluted into autosampler inserts (Thermo Scientific), dried in a speedvac and reconstituted with 5% acetonitrile, 5% formic acid for MS analysis.

#### Tandem Mass Spectrometry and Data Analyses

Mass spectrometric data were collected on an Orbitrap Eclipse mass spectrometer coupled to a Proxeon NanoLC-1000 UHPLC (Thermo Fisher Scientific). The 100 μm capillary column was packed in-house with 35 cm of Accucore 150 resin (2.6 μm, 150 Å; ThermoFisher Scientific). Data were acquired for 180 min per run. The scan sequence began with an MS1 scan collected in the orbitrap (Resolution – 60,000; Scan range – 400−1600 Th; automatic gain control (AGC) target – 4e^5^; normalized AGC target – 100%; maximum injection time – 50 ms. MS2 scans were collected in the orbitrap after higher-energy collision dissociation (Resolution – 50,000; AGC target – 1.25e^5^; normalized AGC target – 250%, NCE (normalized collision energy) – 36, isolation window – 0.5 Th; maximum injection time – 100 ms; TopSpeed set at 1 second). FAIMS compensation voltages (CVs) were set at -40 V, -60 V, and -80 V. Database searching included all entries from the mouse UniProt Database (downloaded in May 2021). The database was concatenated with one composed of all protein sequences for that database in the reversed order.^56^ Raw files were converted to mzXML, and monoisotopic peaks were re-assigned using Monocle.^57^ Searches were performed with Comet^58^ using a 50-ppm precursor ion tolerance and fragment bin tolerance of 0.02. TMTpro labels on lysine residues and peptide N-termini +304.207 Da), as well as carbamidomethylation of cysteine residues (+57.021 Da) were set as static modifications, while oxidation of methionine residues (+15.995 Da) was set as a variable modification. Peptide-spectrum matches (PSMs) were adjusted to a 1% false discovery rate (FDR) using a linear discriminant after which proteins were assembled further to a final protein-level FDR of 1% analysis. TMT reporter ion intensities were measured using a 0.003 Da window around the theoretical m/z for each reporter ion. Proteins were quantified by summing reporter ion counts across all matching PSMs. More specifically, reporter ion intensities were adjusted to correct for the isotopic impurities of the different TMTpro reagents according to manufacturer specifications. Peptides were filtered to exclude those with a summed signal-to-noise (SN) < 180 across all TMT channels and < 0.5 precursor isolation specificity. The signal-to-noise (S/N) measurements of peptides assigned to each protein were summed for a given protein.

#### Cross-linking Assays

Since C4orf3/ALN lacks Cys residues, we introduced a W48C substitution for the Cys cross-linking study, following previous research on SLN.^23^ Mouse C4ORF3/ALN^W48C^ was cloned into the pMSCV-blasticidin vector (75085; Addgene) using gBlocks Gene Fragments (Integrated DNA technologies) tailored to the protein coding sequence. Constructs were confirmed by sequencing and retrovirus was produced in HEK293 packaging cells using 10 μg of plasmid and 5 μg each of the packaging plasmids VSV and gag-pol via the calcium phosphate transfection method. The viral supernatant was collected and filtered (0.45 μm) 48 h later and the inguinal WAT-derived SVF was infected with virus and 10 μg/mL polybrene for 24 h. Blasticidin (10 μg/mL, A1113903; Thermo Fisher Scientific) was used for selection of stable cells. ALN^W48C^ was then cross-linked to SERCA2b in inguinal WAT-derived adipocytes using the homo-bifunctional sulfhydryl cross-linker bismaleimidohexane (BMH). In a buffer consisting of 40 mM MOPS (pH 7.0), 3.2 mM MgCl_2_, 75 mM KCl and 1 mM EGTA, 15 μg of beige adipocyte microsomes were mixed with 3 mM ATP and 1 mM BMH. To probe ALN binding to SERCA2b in the various kinetic states of the Ca^2+^ pump, chemical analogs were added to the preparation for 45 min at 25°C prior to BMH addition. Cross-linking in the E2 state was achieved by incubating microsomes without ATP before the addition of BMH. To visualize ALN binding in the E2-ATP state, 3 mM ATP was added. The E1Ca_2_ and E1PCa_2_ states were analyzed by adding 100 μM free Ca^2+^ to the reaction mixture without or with 3 mM ATP, respectively. The E2V state, an analog to the E2P state of SERCA, was probed by adding 0.1 mM to the reaction mixture. To study the E1PCa_2_·ADP state, 3 mM ADP, 3 mM KF and 50 μM AlCl_3_ were added to the reaction to create an analogous E1·AlFx·ADP state. Lastly, the E2·AlF_4_ state was analyzed as an analog to E2P hydrolysis by adding 3 mM KF and 50 μM AlCl_3_ to the cross-linking reaction. To study the effect of varying Ca^2+^ concentrations (0-100 μM) on ALN binding to SERCA2b, CaCl_2_ was added to the mixture to obtain the desired free Ca^2+^ concentration as determined by the MAXCHELATOR program. After 1 h of incubation at 25°C, the cross-linking reaction was stopped with Laemmli buffer containing 100 mM dithiothreitol. Cross-linking of ALN to SERCA2b was ascertained by immunoblotting with a band present at ∼120 kDa.

#### Mouse Physiology

For the measurement of fat and lean mass, the 3-in-1 Echo MRI Composition Analyzer (Echo Medical Systems) was used in mice following 13 weeks of chow feeding at 30°C. For the insulin tolerance tests, mice were fasted for three hours and insulin (0.5 U kg^-1^ body weight) was injected prior to the collection of blood samples for glucose measurements at 15, 30, 60, 90, and, 120 min post-injection using blood glucose test strips (Freestyle Lite). For cold exposure studies, eight-week-old CRISPRi-*C4orf3* mice mice and littermate controls were housed at thermoneutrality (30°C) for 1 week prior to cold tolerance at 6°C for 9 h. To improve cold acclimation, *Ucp1-/-* x CRISPRi-*C4orf3* mice and littermate *Ucp1-/-* controls at 8 weeks old were given 1mg/kg CL316,243 via i.p. injection for 5 days prior to cold exposure (6°C for 6 h). Rectal temperatures were monitored hourly using a TH-5 thermometer (Physitemp). Mouse forearm grip strength was measured by placing front limbs of mice on a metal grid and maximal peak force was recorded from a median of 5 trials (Ametek, Chatillon).

#### RNA-sequencing and Data Analysis

Total RNA was purified using TRIZOL (ThermoFisher) and RNeasy mini kit (Qiagen) with DNase (Qiagen) treatment on column. Total RNA samples were quantified using Qubit 2.0 Fluorometer (Life Technologies, Carlsbad, CA, USA) and RNA integrity was checked with the 4200 TapeStation (Agilent Technologies, Palo Alto, CA, USA). Samples were initially treated with TURBO DNase (Thermo Fisher Scientific, Waltham, MA, USA) to remove DNA contaminants. ERCC RNA Spike-In Mix (ThermoFisher) was added to normalized total RNA prior to library preparation following manufacturer’s protocol. The next steps included performing rRNA depletion using QIAGEN FastSelect rRNA HMR Kit (Qiagen). RNA sequencing libraries were constructed with the NEBNext Ultra II RNA Library Preparation Kit for Illumina according to manufacturer’s instructions. The sequencing libraries were multiplexed and clustered on the flowcell. After clustering, the flowcell was loaded on the Illumina NovaSeq instrument. The samples were sequenced using a 2x150 Paired-End (PE) configuration. After demultiplexing, sequence data was checked for overall quality and yield. Then, raw sequence reads were trimmed to remove possible adapter sequences and nucleotides with poor quality using Trimmomatic v.0.36. The reads were then mapped to the reference genome (mm10) using the STAR aligner v.2.5.2b. Unique gene hit counts were calculated by using feature Counts from the Subread package v.1.5.2. Only unique reads that fell within exon regions were counted. Downstream analysis was performed on R. Using edgeR, a comparison of gene expression between the groups of samples was performed. Genes with a false-discovery rate of less than 0.05 were called as differentially expressed genes. Gene ontology analysis was performed using ClusterProfiler. A heatmap was drawn using pheatmap on R.

#### RT-qPCR

Trizol (Invitrogen) was used to isolate total RNA from mouse tissue while the iScript cDNA synthesis kit (Bio-Rad) was used for reverse transcription according to manufacturer instructions. PCR reactions were performed on the QuantStudio 6 Flex Real Time PCR System (Applied Biosystems) using Sybrgreen (Bio-Rad). Assays were performed in duplicate, and 36B4 expression was used as an internal control. For human studies, adipose tissue was homogenized in Qiazol and mRNA extracted from tissue using the RNeasy Lipid Kit (Qiagen). The Qiagen Quantitect reverse transcription kit was used to synthesize cDNA. PCR reactions were performed in triplicate using a Roche Lightcycler 480, using gene specific primers (Invitrogen Ltd) and SYBR Green, or with Taqman assays. Transcript levels are presented as the ratio of the abundance of the respective gene of interest: mean abundance of control genes (*PPIA*, *RNA18S5* and *RPLP0*). The PCR primer sequences used to amplify all target genes are provided in Table S2.

#### Tissue Histology and Image Acquisition

Adipose tissues were fixed in 4% paraformaldehyde overnight at 4°C, followed by dehydration in 70% ethanol. They were then embedded in paraffin and cut into sections at a thickness of 5 μm. Sections were stained with haematoxylin and eosin according to the standard protocol at the Beth Israel Deaconess Medical Center pathology core. All light microscopy images were taken with an Axioimager Upright M1 Epifluorescence and Brightfield Microscope (Zeiss) using the 20X Apochromat objective. Five representative images were taken for each inguinal and epididymal white adipose tissue sample. A scale bar (100 μm) is added to each image. All adipose tissue microscopy images were imported into ImageJ software and analyzed using the following protocol: The scale was first set using the scale bar added during image acquisition to convert pixels to micrometers. The image threshold was adjusted to monochrome scale (black and white) so that the adipocyte border was clear. Each intact adipocyte was selected using the wand/tracing tool (with adipocytes on the edge excluded), recorded in the ROI manager, and area was measured using the measure function. Data was recorded and analyzed in Prism. In total, 1770 eWAT cells and 3439 iWAT cells were counted.

#### Electromyography

Prior to the assay, mice were restrained and anesthetized with a 1.2% working solution of tribromoethanol. Twenty-nine-gauge needle electrodes were then placed near the latissimus dorsi muscles in the back. The EMG signal was processed (low-pass filter, 3 kHz; high-pass filter, 10 Hz; notch filter, 60 Hz) and amplified with Bio Amp (1,000 x; ADInstruments, Colorado Springs, CO). Using LabChart 9 Pro software (ADInstruments), EMG data was collected from the electrodes at a sampling rate of 2 kHz, and the raw signal was converted to root mean square (RMS) activity. The data was analyzed for muscle shivering in 10 s windows.

#### Measurement of Triglycerides

Liver triglyceride content was measured according to the manufacturer’s recommendations (ab65336; Abcam).

#### Insulin Measurement

Serum insulin concentrations were measured according to the manufacturer’s recommendations (EZRMI-13K; Millipore).

### Quantification and statistical analysis

#### Statistics

Data were expressed as mean ± s.e.m. Statistical analysis was performed using GraphPad Prism 8 (GraphPad Software, Inc., La Jolla, Ca). *P value*s below 0.05 were considered statistically significant throughout the study. When two-group comparisons were performed, a two-sample unpaired Student’s t-test was used. For Figure 4B, the Mann-Whitney U-test was applied. Welch’s test was used for samples with unequal variance. One-way ANOVA followed by Tukey’s post hoc HSD test was used for multiple group comparisons. Two-way ANOVA with Šídák's multiple comparisons test was used for cold and insulin tolerance tests. Histology of Epi WAT and Ing WAT was performed blinded.

## Supplemental information

Document S1. Figures S1–S6 and Table S2

Table S1. Genes upregulated selectively in the inguinal WAT of DKO mice

Data S1. C4orf3 supplementary material

Document S2. Article plus supplemental information
