# Supplementary figures and images for: Identification of a molecular resistor that controls UCP1-independent Ca2+ cycling thermogenesis in adipose tissue
Source: Cell Metab. 2025 Jun 3;37(6):1311–1325.e9. doi: 10.1016/j.cmet.2025.03.009 (PMC12137002; doi:10.1016/j.cmet.2025.03.009)

Fig. 1F

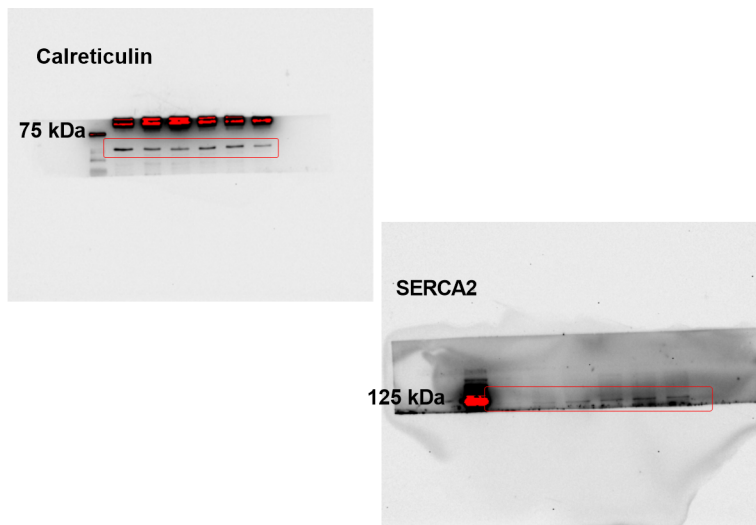

Fig. 2D

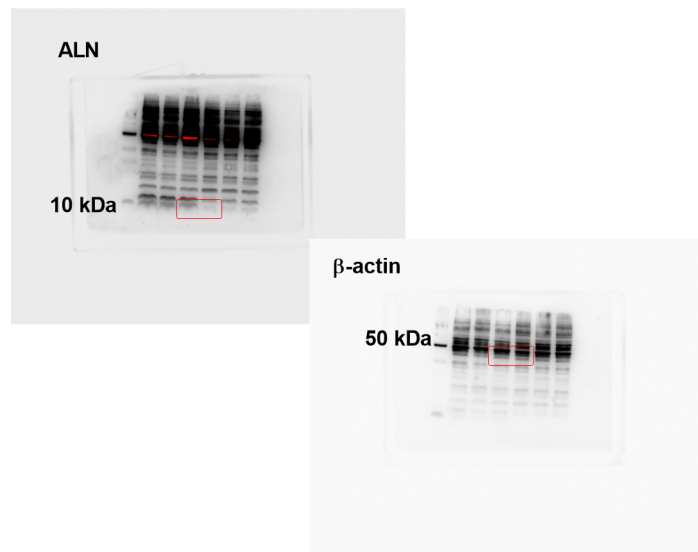

Fig. 2C

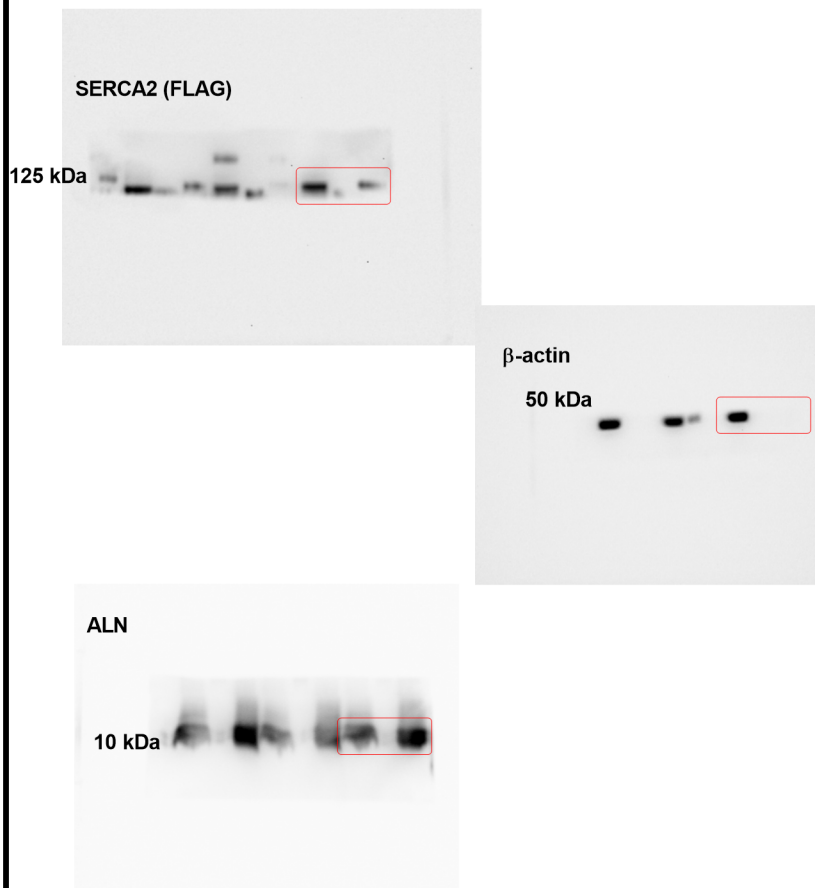

Fig. 3C

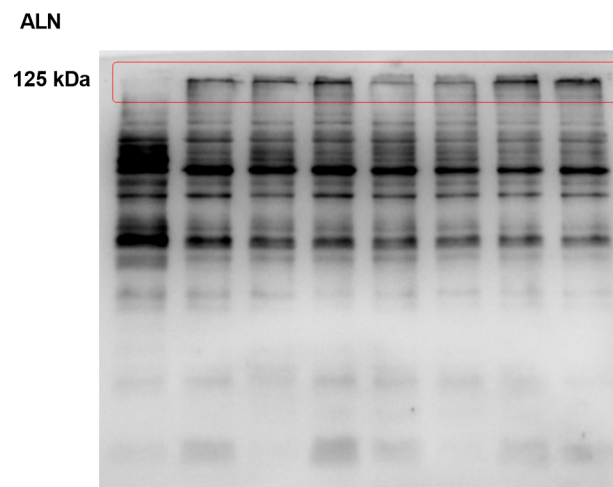

Fig. S2C

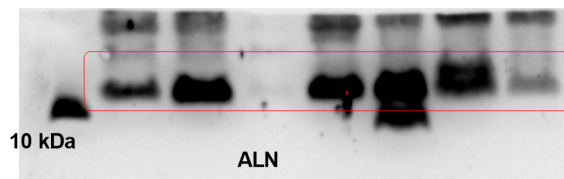

Fig. S2G

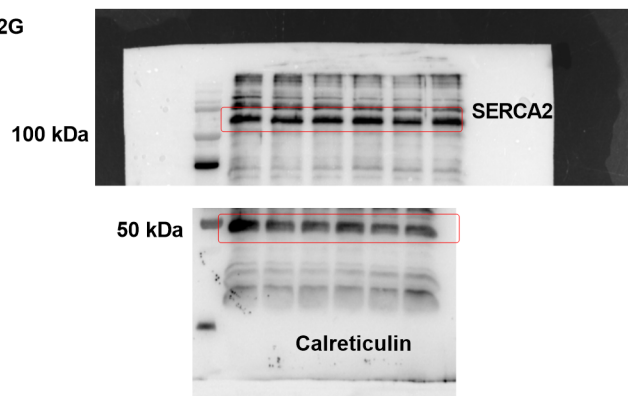

Fig. S3E

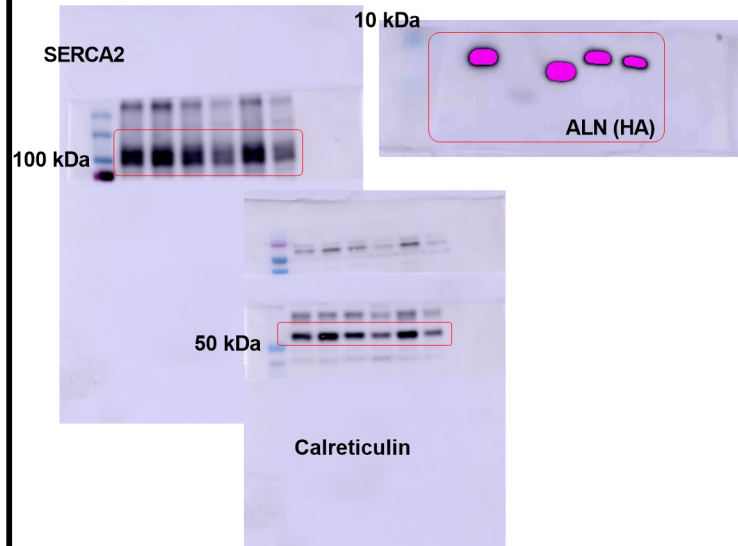

Fig. S4D

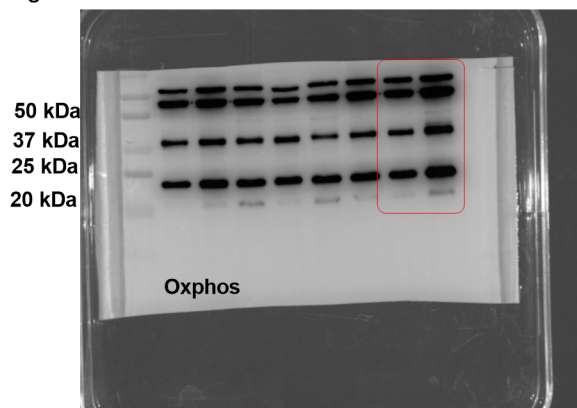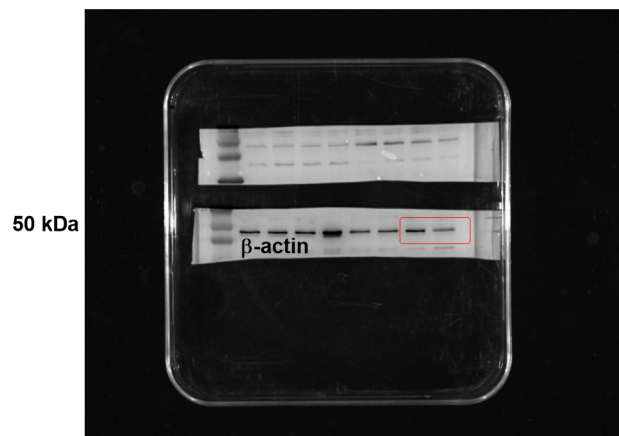

Fig. S4F

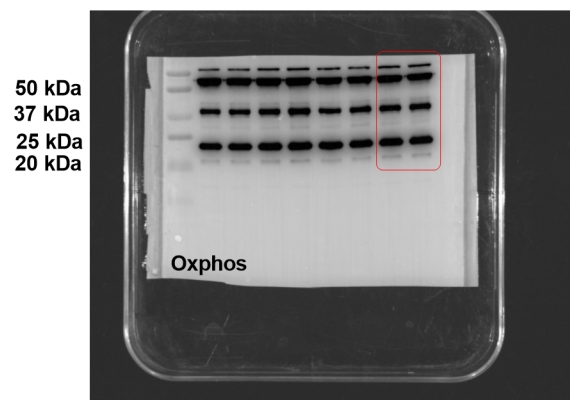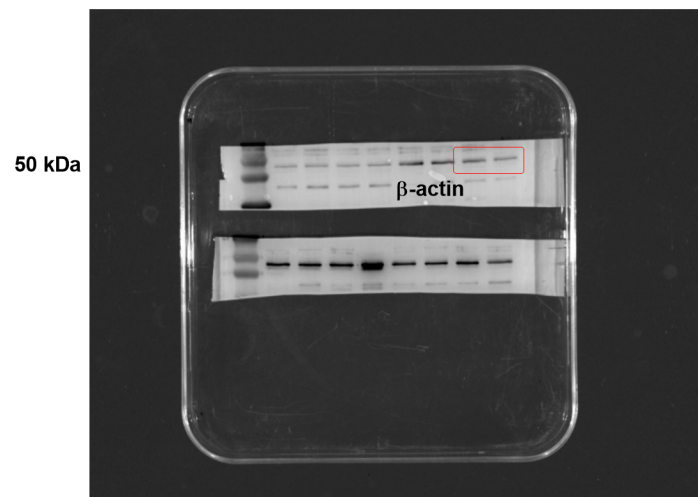

Fig. 2B

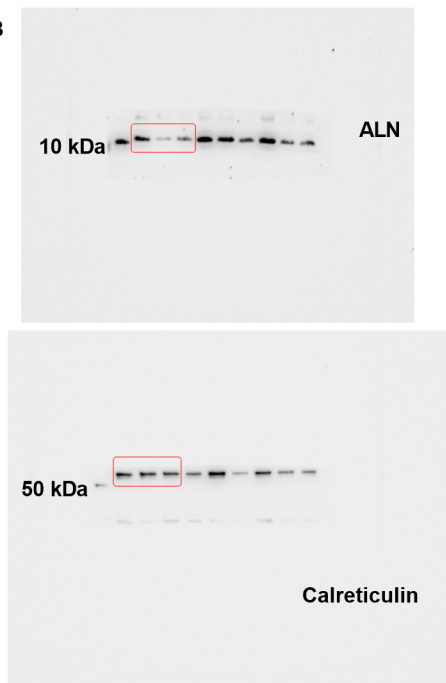

Fig. S4C

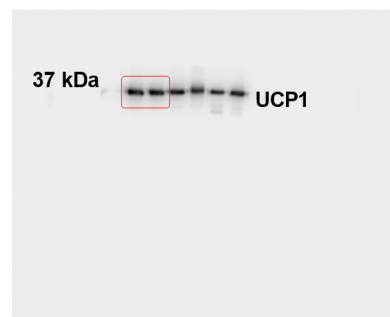

Fig. S4F

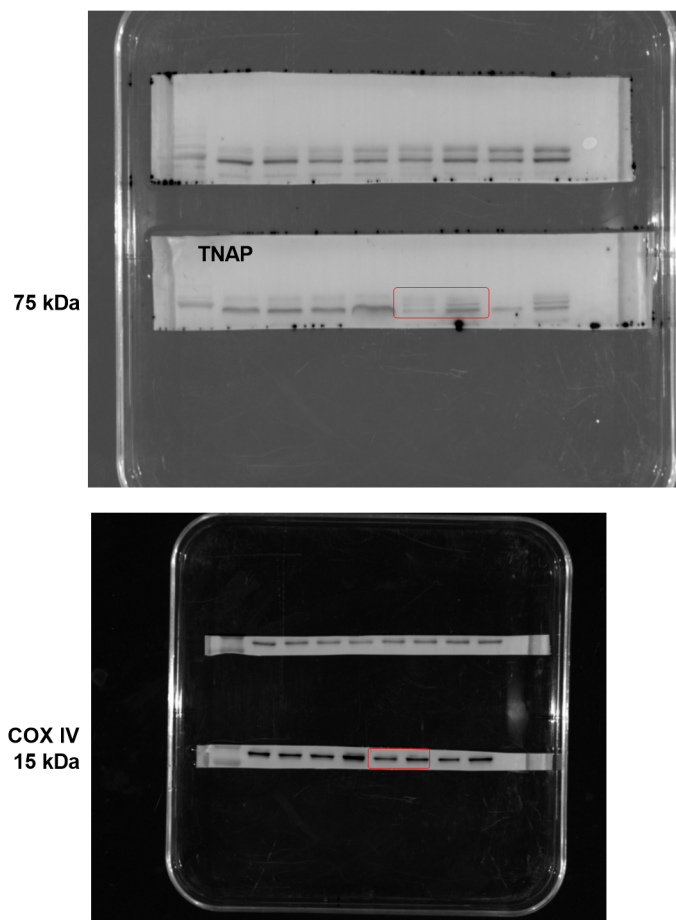

Supplement: Data S1. C4orf3 supplementary material [file mmc3.zip › Source File_S1_02202025/Uncropped Western Blots.pdf]

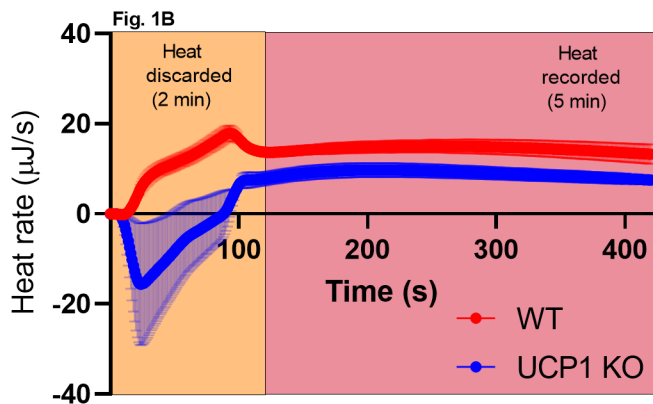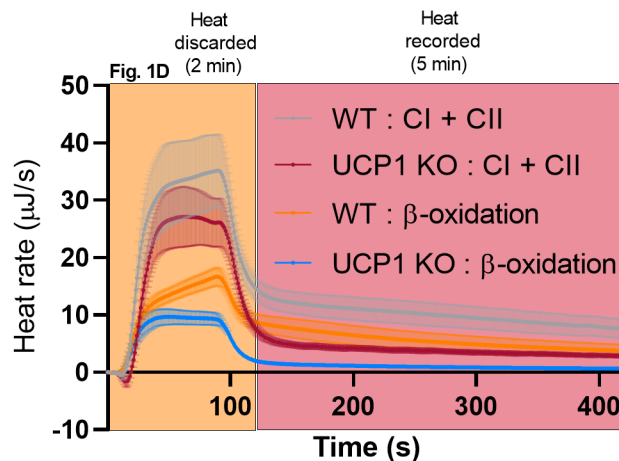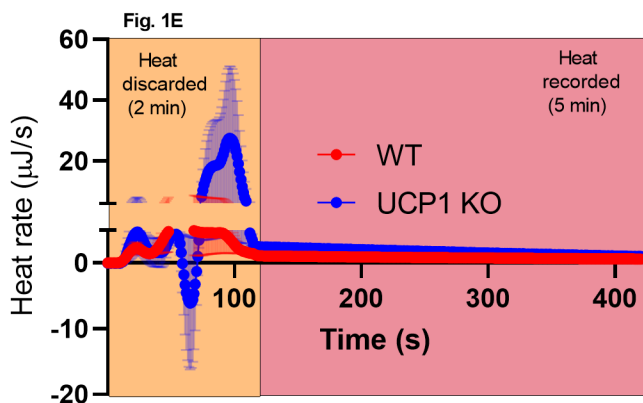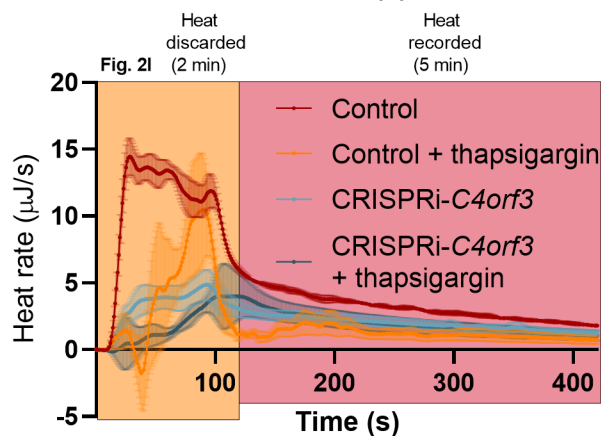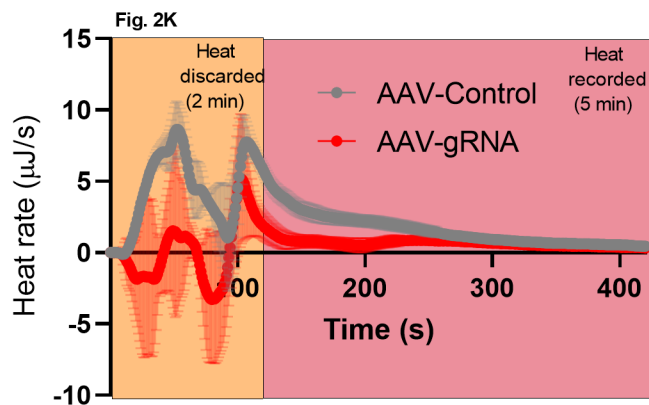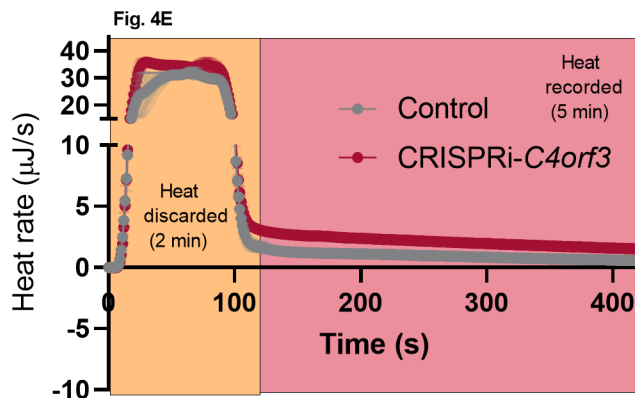

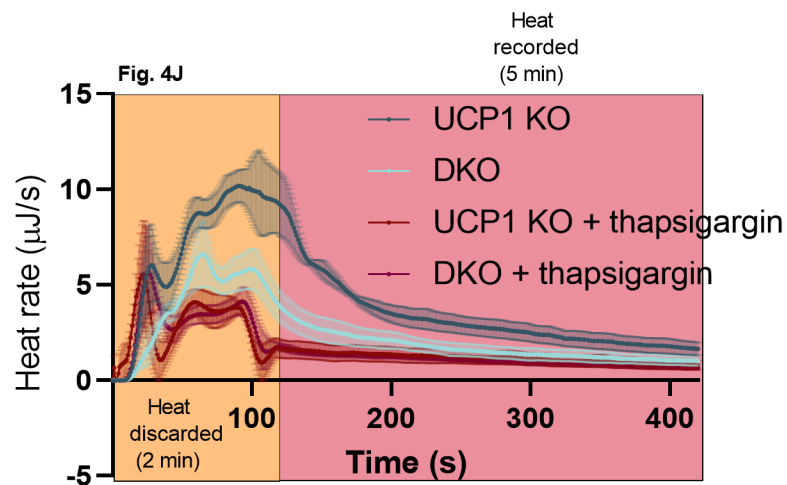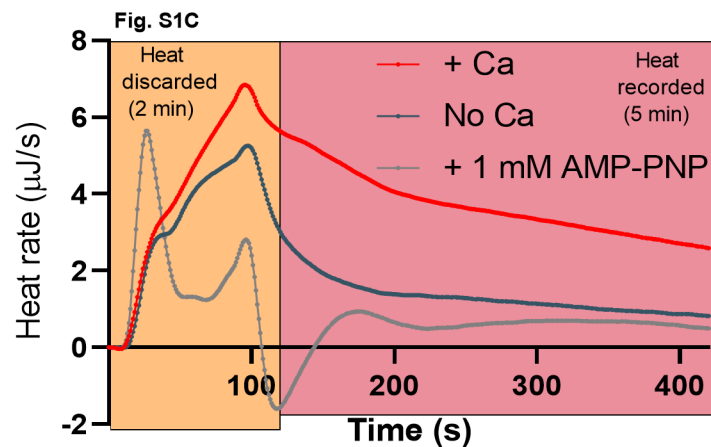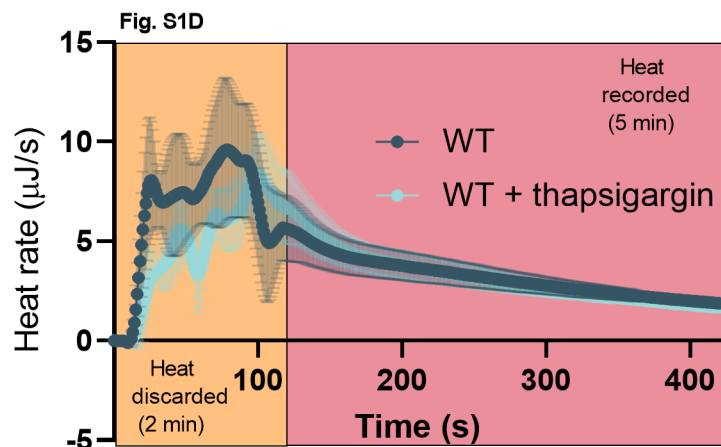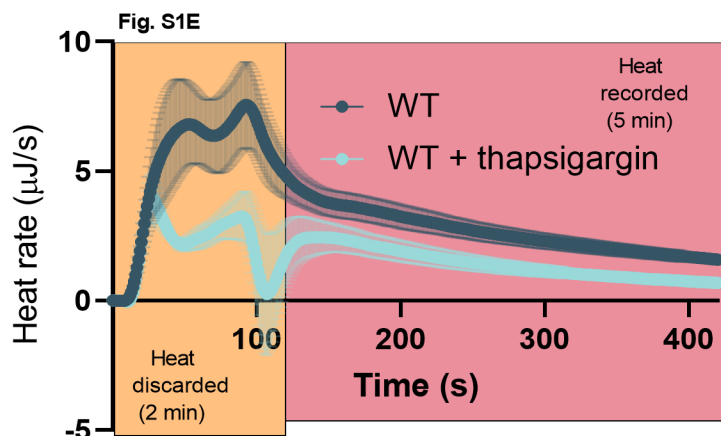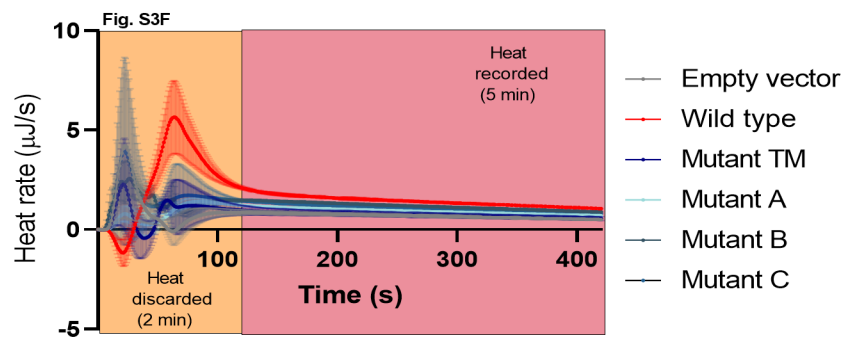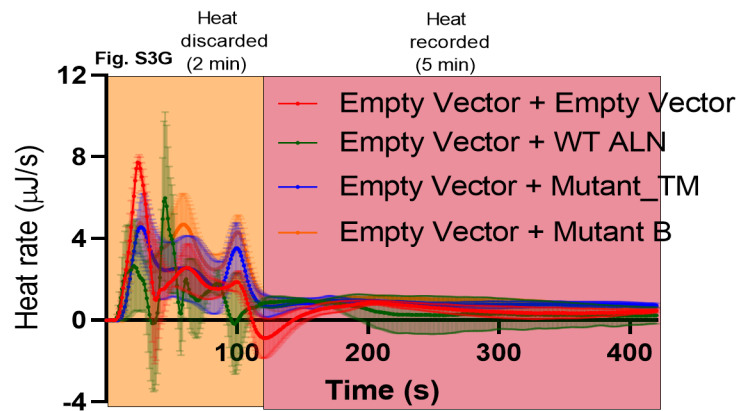

Supplement: Data S1. C4orf3 supplementary material [file mmc3.zip › Source File_S1_02202025/Full ITC graphs.pdf]
