# Supplementary material for: Identification of a molecular resistor that controls UCP1-independent Ca2+ cycling thermogenesis in adipose tissue
Source: Cell Metab. Author manuscript; Available in PMC 2025 Jun 5. (PMC12137002; doi:10.1016/j.cmet.2025.03.009)
Supplement: Supplementary file 1 [file NIHMS2073926-supplement-Sup_Figures.pdf]

Cell Metabolism, Volume *37*

Supplemental information

Identification of a molecular resistor that controls UCP1-independent Ca^{2+} cycling thermogenesis in adipose tissue

Christopher Auger, Mark Li, Masanori Fujimoto, Kenji Ikeda, Jin-Seon Yook, Timothy R. O'Leary, María Paula Huertas Caycedo, Cai Xiaohan, Satoshi Oikawa, Anthony R.P. Verkerke, Kosaku Shinoda, Patrick R. Griffin, Kenji Inaba, Roland H. Stimson, and Shingo Kajimura

A

Ing WAT

Protein	FPKM WT	FPKM UCP1 KO
**Endoregulin**	0.50 ± 0.06	0.36 ± 0.16
**DWORF**	0.00 ± 0.00	0.00 ± 0.00
**Myoregulin**	0.18 ± 0.09	0.69 ± 0.15
**Sarcolipin**	0.28 ± 0.28	0.12 ± 0.13
**Phospholamban**	0.45 ± 0.02	0.64 ± 0.05

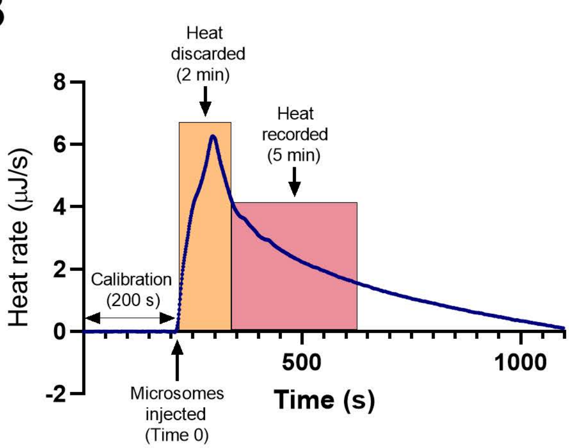

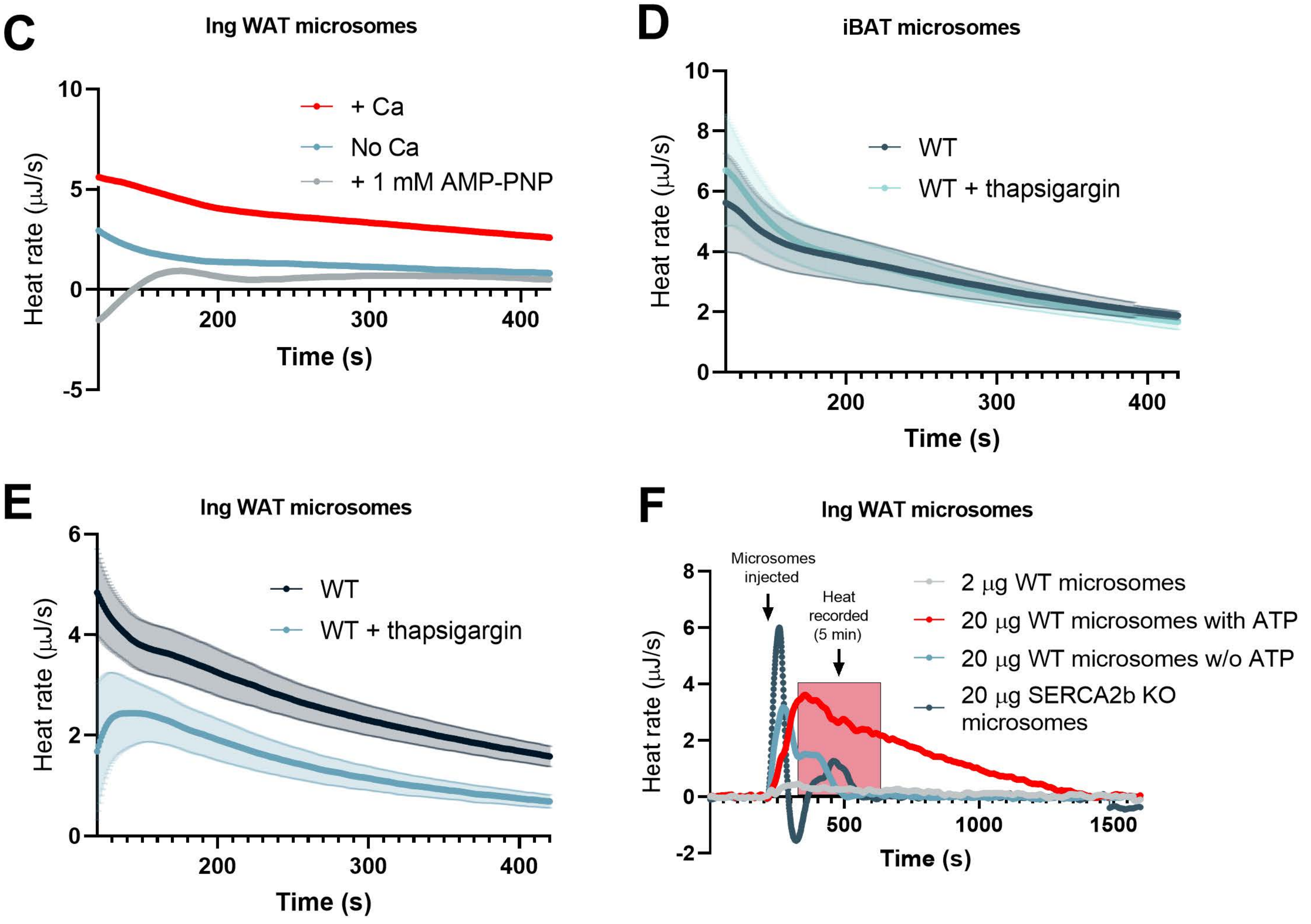

Supplementary Figure 1

Figure S1. Direct measurement of microsomal thermogenesis (Related to Figure 1)

A. mRNA expression levels (FPKM) of indicated SERCA-binding peptides in the inguinal WAT of wild-type and UCP1 KO mice. The data were obtained from E-MTAB-4085.

B. Representative ITC graph from microsomes of inguinal WAT demonstrating how heat is quantified from biological material. Microsomal thermogenesis was recorded during the indicated time following the addition of Ca^{2+} in the presence of ATP.

C. Heat rate ($\mu J\ sec^{-1}$) in isolated microsomes from the inguinal WAT of wild-type mice. Microsomal thermogenesis was recorded in the presence or absence of free Ca^{2+} or 1 mM AMP-PNP in place of 1 mM ATP.

D. Heat rate ($\mu J\ sec^{-1}$) in isolated microsomes from the interscapular BAT of wild-type mice. Microsomal thermogenesis was recorded during the indicated time following the addition of ATP and free Ca^{2+} in the presence or absence of 15 µM thapsigargin. n = 4 per group.

E. Heat rate ($\mu J\ sec^{-1}$) in isolated microsomes from the inguinal WAT of wild-type mice. Microsomal thermogenesis was recorded during the indicated time following the addition of ATP and free Ca^{2+} in the presence or absence of 15 µM thapsigargin. n = 4 per group.

F. Heat rate ($\mu J\ sec^{-1}$) in isolated microsomes (2 µg or 20 µg) from the inguinal WAT of wild-type mice and fat-specific SERCA2 KO mice (Adipo-Cre x *Atp2a2*$^{flox/flox}$ mice). Microsomal thermogenesis was recorded during the indicated time following the addition of free Ca^{2+} in the presence or absence of ATP.

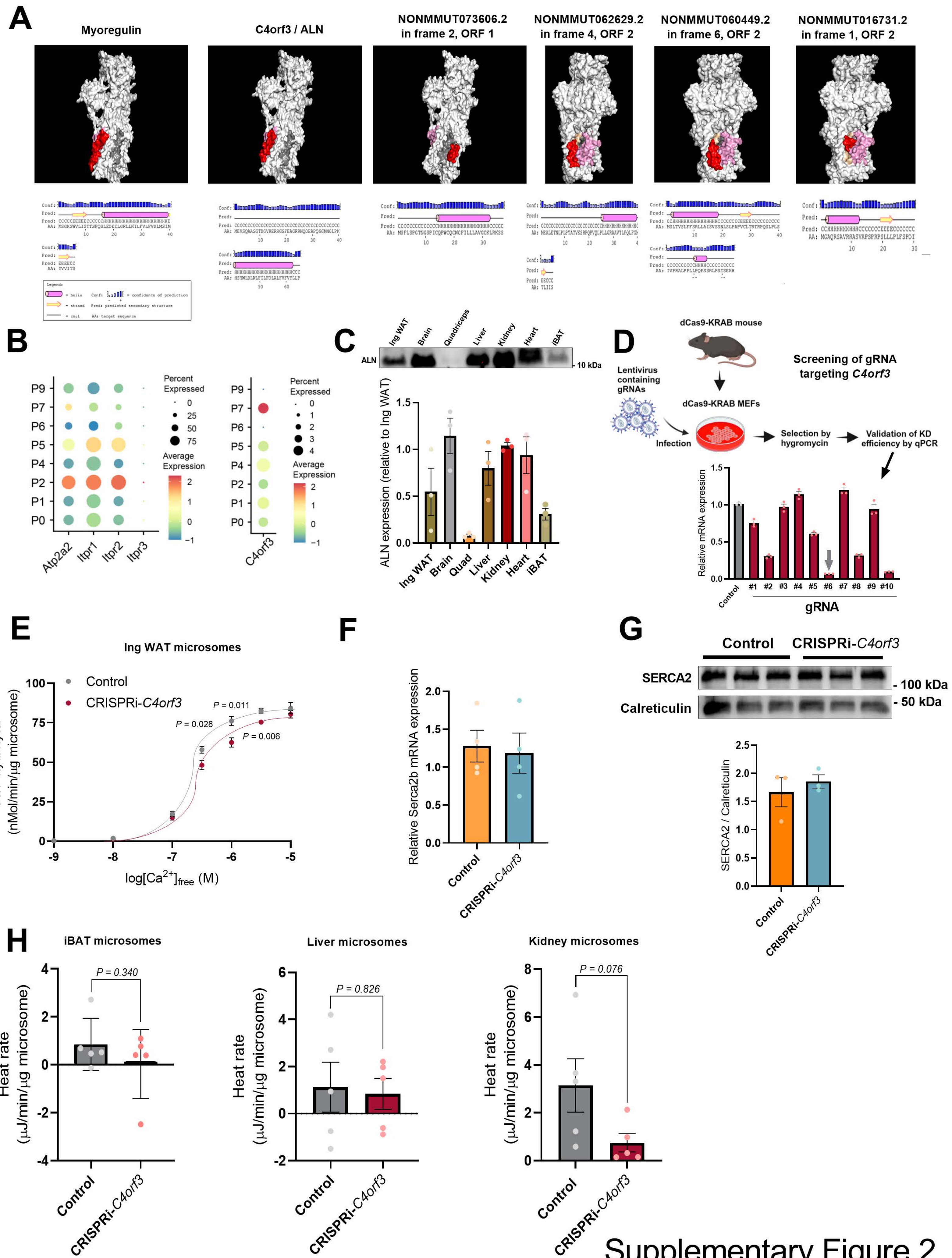

Supplementary Figure 2

Figure S2. Identification of C4orf3 as a SERCA2-binding peptide (Related to Figure 2)

A. Top: 3D structural interaction models of indicated candidates and SERCA1 using I-TASSER. The HDOCK server was used for the docking simulations, with all parameters set to default values. Myoregulin is a known SERCA1 binding partner and used as a positive control for the prediction. Bottom: amino acid sequence of each candidate and the location of the predicted transmembrane domain.

B. Single-cell expression data of indicated genes involved in Ca^{2+} cycling thermogenesis (*Atp2a2*, *Itpr1*, *Itpr2*, *Itpr3*, and *C4orf3*) in UCP1 KO inguinal white adipose tissue (WAT). Data were obtained from single-nucleus RNA-seq of inguinal WAT from *Ucp1* KO mice after cold exposure at 8°C [33].

C. Relative ALN protein expression in isolated microsomes from indicated organs of wild-type mice kept at room temperature. Equal amounts of microsomes (20 ug) were loaded into the SDS gel for immunoblotting. n = 3.

D. Screening of effective gRNAs targeting *C4orf3*. Mouse embryonic fibroblasts (MEF) originated from dCas9-KRAB-derived mice was used to search for effective gRNAs that suppress *C4orf3* mRNA expression. qPCR analyses of the MEFs identified gRNA#6 (5'-TTGGCGGGGTTA CCCGGAAT-3') was the most effective gRNA. n = 3 per group.

E. SERCA ATPase assay in isolated microsomes from the inguinal WAT of CRISPRi-*C4orf3* mice and littermate control mice. ATP hydrolysis activities were measured at indicated free Ca^{2+} concentrations. n = 5 per group. Statistic: unpaired two-tailed t-test.

F. Relative mRNA expression of *Serca2b* in the inguinal WAT of control and CRISPRi-*C4orf3* mice. n = 4 per group.

G. SERCA2 protein expression in isolated microsomes from control and CRISPRi-*C4orf3* mice. Calreticulin was used as a loading control. n = 3 per group.

H. Heat rate ($\mu J\ sec^{-1}$) in isolated microsomes from the iBAT, the liver and kidney of control and *C4orf3*^{CRISPRi} male mice. Microsomal thermogenesis was recorded during the indicated time following the addition of ATP and free Ca^{2+}. Thapsigargin was added to calculate SERCA-dependent thermogenesis. Note that there was no detectable SERCA-dependent (thapsigargin-sensitive) heat production in the iBAT and liver. Quantifications of heat rate normalized by microsome protein contents ($\mu J\ min^{-1}\ \mu g^{-1}$) were shown. n = 5 per group. Statistic: unpaired t-test. Bars represent mean and error, as shown in s.e.m.

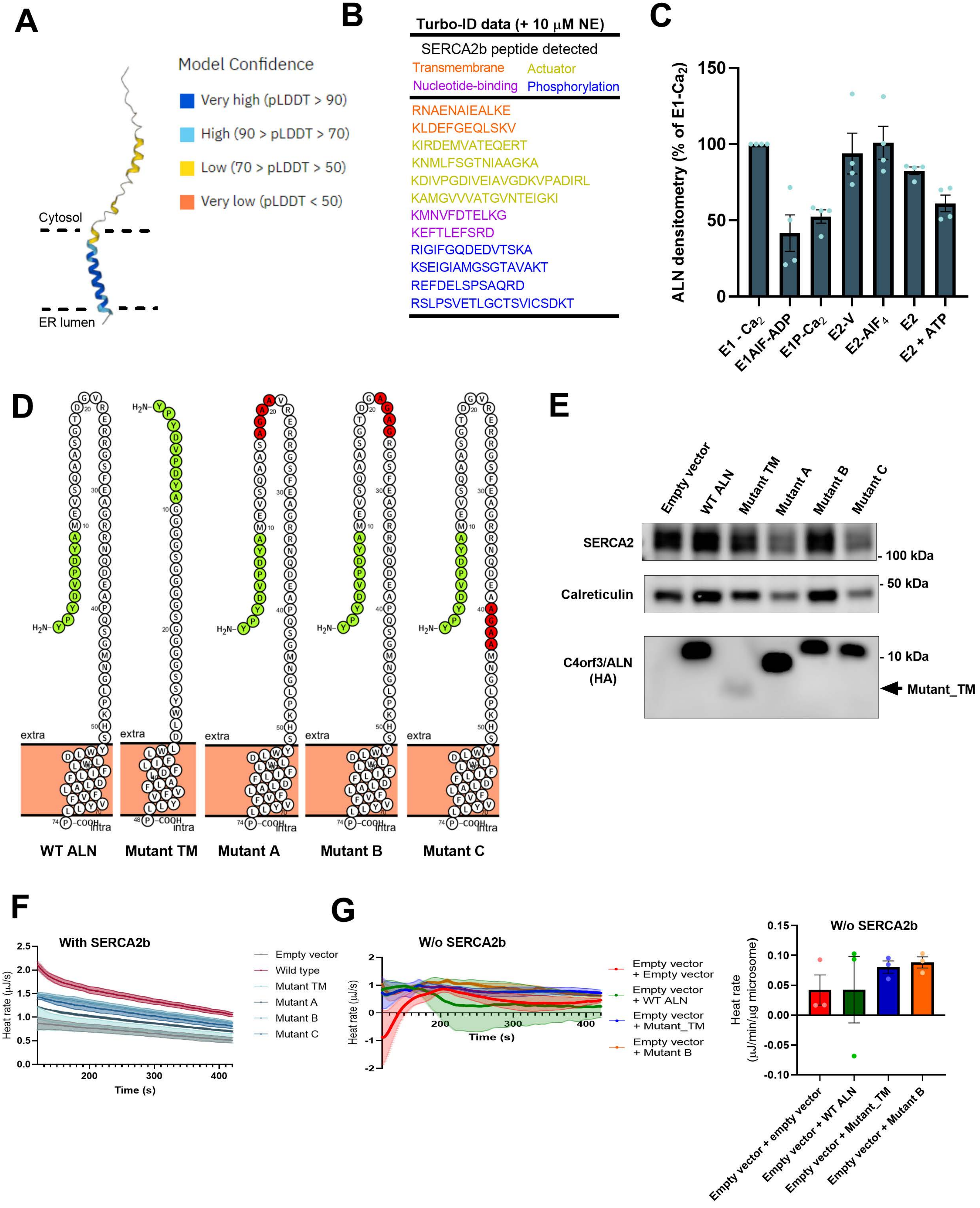

Supplementary Figure 3

Figure S3. Interaction between C4orf3/ALN and SERCA2b protein (Related to Figure 3)

A. Predicted structure of C4ORF3 protein by AlphaFold3. The indicated colors represent the model confidence (pLDDT).

B. SERCA2b peptide sequences identified by the Turbo-ID proximity-labeling proteomics in the presence of norepinephrine. Cells were stimulated with norepinephrine at 10 μM for 30 min.

C. Quantification of the SERCA2b-C4orf3/ALN^{W48C} interaction from the cross-linking experiment shown in Figure 3C. Densitometry was performed using ImageJ for Windows. n = 4 independent experiments.

D. The amino acid sequence and topology of full-length (WT) C4orf3 and indicated mutants.

E. Immunoblotting of SERCA2 and indicated C4orf3 mutants. The antibody for calreticulin was used as a loading control. Molecular weight (kDa) is shown on the right.

F. Heat rate (μJ sec^{-1}) in isolated microsomes from HEK293 cells stably expressing an empty vector, full-length (WT) C4orf3, and indicated C4orf3 mutants, and transiently expressing SERCA2b. Microsomal thermogenesis was recorded during the indicated time following the addition of Ca^{2+} and ATP. n = 3 per group.

G. Heat rate (μJ sec^{-1}) in isolated microsomes from HEK293 cells stably expressing an empty vector, full-length (WT) C4orf3, and indicated C4orf3 mutants, but lacking exogenous SERCA2b expression. Microsomal thermogenesis was recorded during the indicated time following the addition of ATP and free Ca^{2+}. Right: Microsomal heat rate was normalized by microsome protein contents (μJ min^{-1} μg^{-1}). n=3 per group. Bars represent mean and error, as shown in s.e.m.

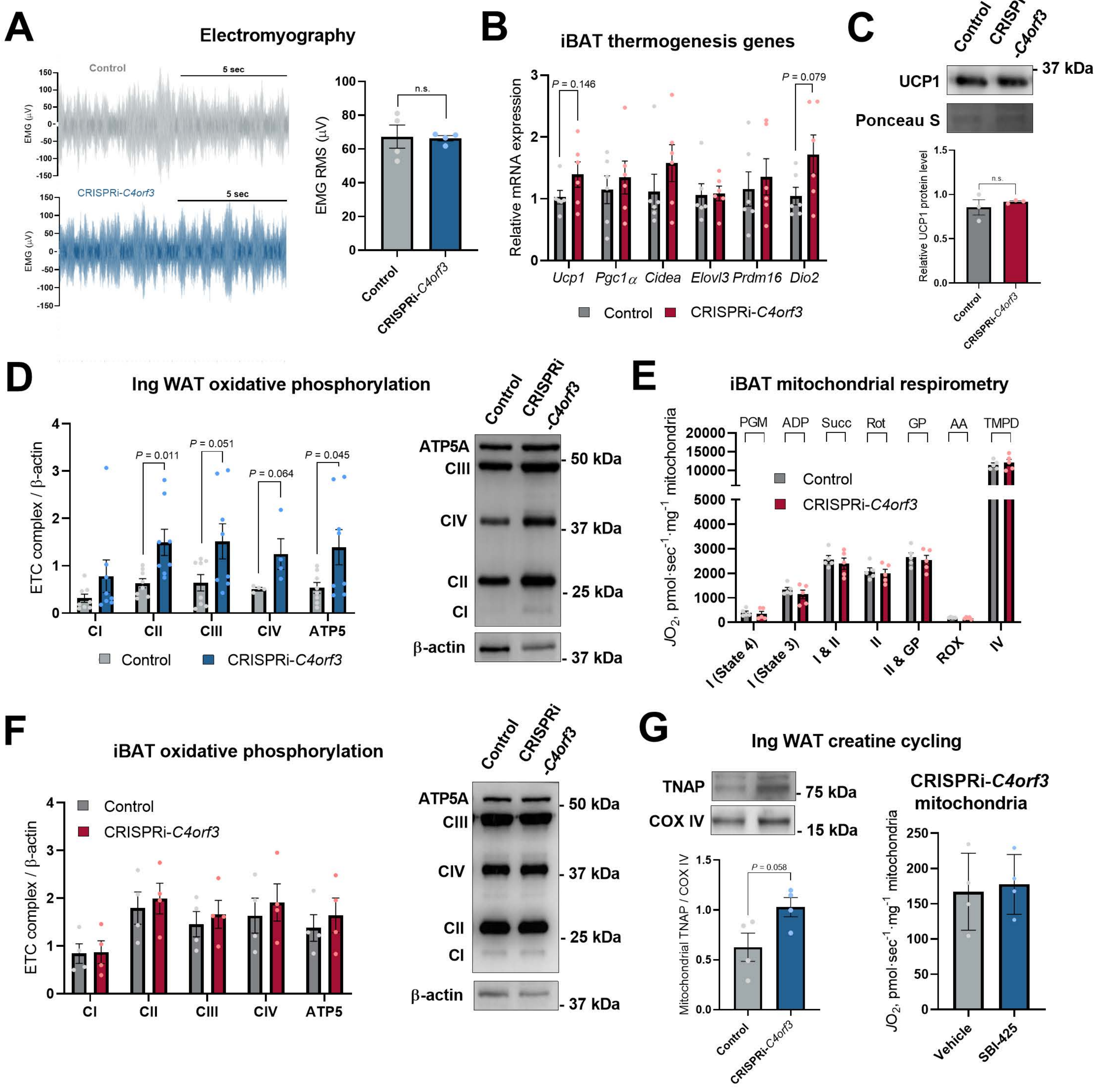

Supplementary Figure 4

Figure S4. Compensatory regulation of UCP and UCP1-independent thermogenic pathways in adipose tissue (Related to Figure 4)

A. Representative electromyography (EMG) traces in the skeletal muscle and the quantification. Male *C4orf3*^{CRISPRi} mice and littermate control mice were challenged to 6˚C cold temperature. EMG recording data were converted to the root mean square (RMS). n = 4 per group. Statistic: unpaired t-test.

B. Relative mRNA expression of indicated thermogenic genes in the iBAT of male *C4orf3*^{CRISPRi} mice and control mice following cold exposure. n = 6 per group. Statistic: two-tailed unpaired t-test.

C. Relative expression levels of UCP1 protein from the iBAT of cold exposed (6˚C for 72 h) control and CRISPRi-*C4orf3* mice. n = 3 per group. Ponceau staining was shown as a loading control. Statistic: unpaired t-test.

D. Relative expression levels of mitochondrial OXPHOS proteins in the inguinal WAT of male *C4orf3*^{CRISPRi} mice and control mice following cold exposure. Expression levels of each complex protein were normalized to β-actin. Representative immunoblotting for mitochondrial OXPHOS proteins is shown on the right. n = 8 per group. Statistic: two-tailed unpaired t-test.

E. Mitochondrial respiration (JO_2) in the iBAT of male *C4orf3*^{CRISPRi} mice and control male mice following cold exposure (6˚C for 72 h). JO_2 at indicated states was measured using the following protocol. For Complex I-driven respiration, 10 mM glutamate, 2 mM malate and 5 mM pyruvate were added. ADP at 4 mM was used to stimulate state 3 respiration. Succinate at 10 mM was added to measure the activity of complex II. Rotenone at 10 µM was used to inhibit complex I. The glycerophosphate pathway was probed with 10 mM rac-glycerol 1-phosphate (GP). Antimycin A (AA) at 2.5 µM was used to inhibit mitochondrial respiration. n = 5 per group.

F. Relative expression levels of mitochondrial OXPHOS proteins in the iBAT of male *C4orf3*^{CRISPRi} mice and control male mice following cold exposure (6˚C for 72 h). Expression levels of each complex protein were normalized to β-actin. Representative immunoblotting for mitochondrial OXPHOS proteins was shown on the right. n = 4 per group.

G. Left: Mitochondrial TNAP protein expression in the Ing WAT of *C4orf3*^{CRISPRi} mice and control mice following cold exposure. Expression levels of TNAP were normalized to β-actin. Representative immunoblotting was shown. n = 4 per group. Right: The effect of a pharmacological TNAP inhibitor SBI-425 on mitochondrial respiration (JO_2) in the Ing WAT of *C4orf3*^{CRISPRi} mice. JO_2 was normalized by mitochondrial protein contents. n = 4 per group. Statistic: unpaired t-test.

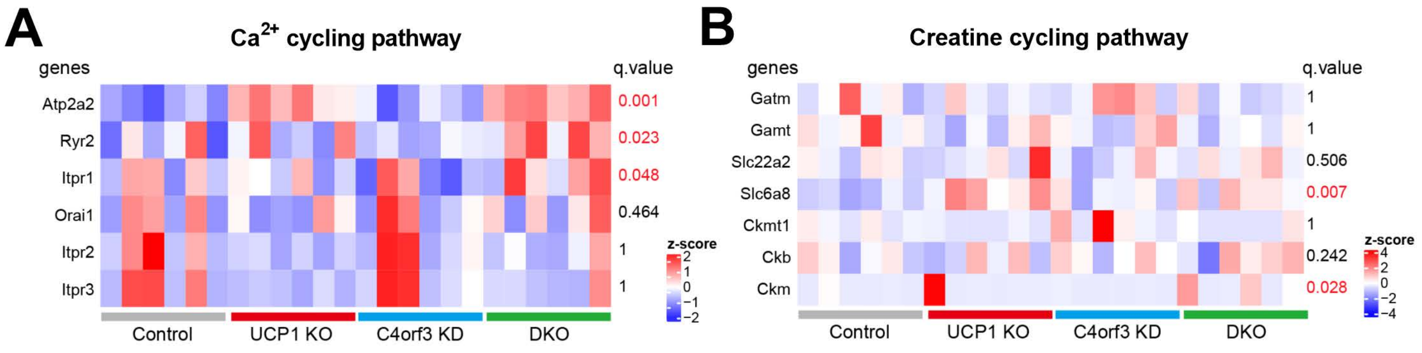

Supplementary Figure 5

Figure S5. Compensatory responses to C4orf3/ALN and UCP1 loss (related to Figure 5)

A and B. Relative mRNA levels of indicated genes in the inguinal WAT of wild-type control mice (control), UCP1 KO mice, *C4orf3*^{CRISPRi} mice, and DKO mice (UCP1 KO x *C4orf3*^{CRISPRi}) following cold exposure at 6 ˚C for 5 hours. Data represented as z-score heat map for each gene in each sample representing quantitated value.

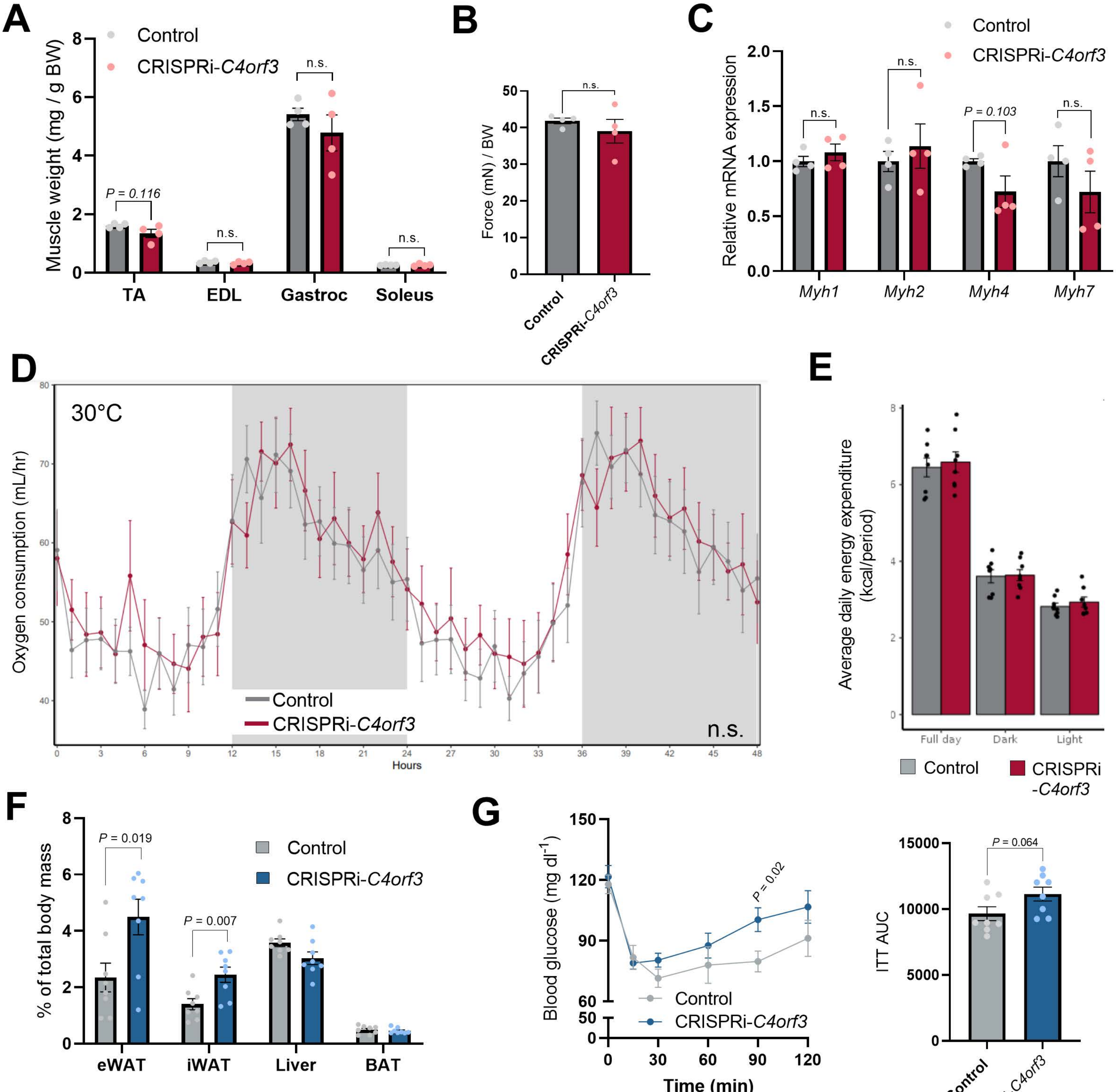

Supplementary Figure 6

Figure S6. Metabolic phenotype of CRISPRi-*C4orf3* mice (Related to Figure 6)

A. Tissue weight of indicated skeletal muscle in CRISPRi-*C4orf3* male mice and littermate control mice. The tissue mass was normalized to body weight. n=4. n.s., not significant.

B. Grip strength normalized to body weight in CRISPRi-*C4orf3* male mice and control mice. n=4.

C. Relative expression of indicated genes involved in muscle fiber type switching in the gastrocnemius muscle of control and CRISPRi-*C4orf3* male mice. Statistic: unpaired t-test. Data represented as mean with s.e.m. n=4.

D. Whole-body oxygen consumption rate of *C4orf3*^{CRISPRi} mice and littermate wild-type control mice at 30˚C. n=7 per group.

E. Quantification of energy expenditure in D. Statistic: Two-way ANOVA with Šídák's multiple comparisons test. Data represented as mean with s.e.m.

F. Indicated tissue mass of female mice on a regular diet at 30˚C. Mice at 20 weeks old were examined. n=8. Stat: two-tailed unpaired t-test. Data represented as mean with s.e.m.

G. Insulin tolerance test and area under the curve (AUC) of female *C4orf3*^{CRISPRi} mice and littermate control mice. Mice kept were fasted for 3 hours prior to collecting baseline blood glucose levels and subsequent injection of insulin (0.5 U per kg body weight). Blood glucose levels were measured at indicated time points. n = 8 per group. Statistic for insulin tolerance curve is 2-way ANOVA with Šídák's multiple comparisons test, and AUC statistic is unpaired t-test. Data represented as mean with s.e.m., individual values presented for AUC.

Supplementary Table 2 qRT-PCR primer sequences.

	Gene	Forward primer	Reverse primer	Taqman ID number
Human	***RNA18S5***	CTTCCACAGGAGGCCTACAC	CGCAAAATATGCTGGAACTTT	
	SERCA2B	ATCTGCCTGTCCATGTCACT	TGTGCTATAGACCCAGATCACC	
	C4ORF3			Hs00386171_m1
Mouse	***36B4***	TCCAGGCTTTGGGCATCA	CTTTATCAGCTGCACATCACTCAGA	
	Aln	GCTGGATCTCTGGCTCTTCA	TCGTTTTCAAGATTCACTCCAG	
	Cidea	ATCACAACTGGCCTGGTTACG	TACTACCCGGTGTCCATTTCT	
	Dio2	CAGTGTGGTGCACGTCTCCAATC	TGAACCAAAGTTGACCACCAG	
	Elovl3	TCCGCGTTCTCATGTAGGTCT	GGACCTGATGCAACCCTATGA	
	Myh1	GCATCCCTAAAGGCAGGCT	AGCCTCGATTCGCTCCTTTT	
	Myh2	CTCGTTTGCCAGTAAGGGTCT	GCCTCGATTCGCTCCTTTTC	
	Myh4	TCTGGTAACACAAGAGGTGC	CATCTCAGCGTCGGAACTCA	
	Myh7	GCCAACTATGCTGGAGCTGATGCCC	GGTGCGTGGAGCGCAAGTTTGTCATAAG	
	Pgc1α	AGCCGTGACCACTGACAACGAG	GCTGCATGGTTCTGAGTGCTAAG	
	Prdm16	GGCGAGGAAGCTAGCCAAA	GGTCTCCTCCTCGGCACTCT	
	Serca2b	AGTTGAGCCAGCAGACATTG	CCAGAGAATCATGCAAAAGACA	
	Ucp1	CTTTATCAGCTGCACATCACTCAGA	CCCTAGGACACCTTTATACCTAATGG	
